# Mental models of the sixth mass extinction reveal pathways for transformative sustainability action

**DOI:** 10.1038/s41598-026-40100-w

**Published:** 2026-02-20

**Authors:** Ganga Shreedhar

**Affiliations:** Department of Psychological and Behavioural Science, London School of Economics and Political Science, London, England

**Keywords:** Extinction, Mental models, Biodiversity loss, System transformation, Policy support, Behaviour change, Ecology, Ecology, Environmental social sciences, Environmental studies, Philosophy, Science, technology and society, Scientific community, Social sciences

## Abstract

To address fundamental challenges to global sustainability posed by unprecedented biodiversity loss and the sixth mass extinction (SME), scientists advocate for transformative changes to systems, policies, and behaviours. Yet public understanding of the biodiversity crisis remains largely unexplored. This article presents the first comprehensive examination of public mental models regarding support for transformative changes using a nationally representative UK survey (n = 739). Whilst only 28% of respondents had heard of the “sixth mass extinction,” 93% accepted the phenomenon once explained, with 95% attributing it to human activities. Principal component analysis revealed distinct mental models for policy support (conservation, market regulation, lifestyle changes, and big technology) and behavioural change (citizenship, consumer actions, waste reduction, and nuclear energy use). Attribution to direct human causes strongly predicted support for transformative change (β = 0.44, *p* < 0.001), while attributions to distant and non-human causes reduced support (β = − 0.20, *p* < 0.001). These findings demonstrate high latent public support for sustainability transformations and provide actionable insights for science communication and policy engagement strategies addressing the biodiversity crisis.

**Supplementary Information:**

The online version contains supplementary material available at 10.1038/s41598-026-40100-w.

## Introduction

The Earth is experiencing biodiversity loss at unprecedented rates, with current species extinction rates estimated to be 100–1000 times faster than the background rate^1,2^. This acceleration has led scientists to warn that we are risking a sixth mass extinction (SME)^1,3,4^. Unlike previous mass extinctions, which were driven by natural catastrophes such as asteroid impacts or massive volcanic eruptions, this crisis stems primarily from human activities including habitat destruction, pollution, overexploitation of natural resources, and anthropogenic climate change^5,6^.

Unchecked biodiversity loss threatens sustainable development and human well-being^6–8^. Biodiversity loss disrupts ecosystem services such as clean air and water, fertile soils, climate regulation, and natural disaster protection, with these with these disruptions cascading through human systems to increase food and water insecurity, heightening climate change and pandemic disease risk, and potentially contributing to social instability and political conflict^8^.

In response to these interconnected threats, major international scientific bodies have issued urgent calls for transformative action. In 2020, the Intergovernmental Science-Policy Platform on Biodiversity and Ecosystem Services (IPBES) called for transformative changes to human institutions and behaviour to halt biodiversity loss^8,9^, conceptualising “transformative change” as encompassing a deep, system-wide reorganization of technological, economic, and social factors, including fundamental shifts in policies, institutions, and underlying behaviours such as consumption, and societal values. The Intergovernmental Panel on Climate Change (IPCC) and United Nations Sustainable Development Goals have similarly emphasised systems transformations that simultaneously address biodiversity loss, climate change, and sustainable development^10–12^.

However, transformative sustainability action fundamentally depends on public understanding, acceptance, and support. System transformations explicitly target several human activities that cause species extinction^8^. Direct human drivers include land-use change, direct exploitation of organisms, climate change, and pollution. Indirect factors emerge from economic, technological, institutional, cultural, and historical factors that underpin direct drivers, such as market incentives that prioritize profits over ecological values, or colonialism. Achieving deep and sustainable transformations requires coordinated changes to address these direct and indirect causes across multiple domains—from individual behaviours and consumption choices to policy frameworks, market structures, and societal values^13,14^. Without broad social backing, policies and interventions may face implementation challenges or outright resistance. Scientists have already warned about the emergence of extinction denial narratives—rhetoric that casts doubt on anthropogenic species extinction—following the release of major IPBES reports^15^. Such narratives could cause confusion and undermine public support for necessary transformative actions, similar to climate change denialism’s impact on climate policy^16^.

Psychological factors including perceptions, beliefs, and mental models of complex environmental problems influence public support for transformative change. Mental models—cognitive and affective representations of systems that capture perceived causal relationships among system components—serve as fundamental psychological frameworks through which individuals understand and respond to complex challenges^17–19^. These models function as sets of causal beliefs that operate in people’s minds, influencing how they perceive the sources and consequences of systemic problems and their willingness to adopt different types of solutions. Mapping mental models is therefore crucial for designing effective sustainability communication and engagement strategies.

Extensive research has examined mental models of climate change, revealing how different causal belief structures lead to distinct patterns of policy and behavioural support^17,19^. For instance, an ‘air pollution’ model of global warming, which attributes climate change primarily to toxic chemicals, tends to support general pollution control measures over specific energy-use reductions. In contrast, a ‘carbon emissions’ model, which focuses on greenhouse gas emissions from fossil fuel use, strongly supports energy transitions and carbon pricing policies^19,20^. These studies demonstrate how the public’s mental models have real-world implications for public engagement and policy implementation.

Despite the critical importance of biodiversity loss for global sustainability, public understanding of extinction has received remarkably little research attention. Biodiversity loss has primarily been studied only as a driver of climate change rather than as a distinct phenomenon requiring its own communication and engagement strategies^19^. No previous study has comprehensively mapped public understanding and perceptions of the sixth mass extinction specifically, despite its designation as one of the most pressing sustainability challenges of our time.

This research gap is particularly notable given that extinction presents distinct challenges compared with climate change^10^. Extinction can involve the permanent loss or degradation of species and ecosystems locally, often with irreversible consequences. The solutions required to address biodiversity loss—such as habitat protection, sustainable resource management, and ecosystem restoration—often differ from those primarily focused on greenhouse gas emissions reduction (although some solutions such as reforestation may also yield climate co-benefits)^8^.

Previous research on environmental mental models has revealed that different belief structures can be in tension with each other, leading to trade-offs in policy support^19–21^. In the climate domain, for example, studies have found that generic ‘green’ beliefs and policy support can be distinct from, and potentially conflict with, more specific ‘carbon emissions’ or ‘geoengineering models^20^. Other research has identified that the public perceives trade-offs between achieving environmental and social sustainability goals^21^. However, these studies have typically focused on limited sets of policies and have not explored how mental models relate to the comprehensive, systems-level transformations to address biodiversity loss called for by IPBES.

This article aims to examine public understanding of the SME and identify mental models of transformative approaches to change, policies, and behaviours. A pre-registered survey was developed using a mental models approach that combined normative research (expert interviews and literature review) with descriptive research (focus groups and surveys)^17^. A nationally representative sample of 739 UK adults was recruited through Prolific Academic in July 2022, with demographic quotas ensuring representativeness by age (M = 44 years), gender (48% male), and ethnicity (87% white).

The survey assessed awareness of the sixth mass extinction, perceived causes and consequences of biodiversity loss, and support for transformative approaches, policies and behaviours. In addition, psychological factors including perceived risk, concern and controllability, trust in science, perceived disagreement among scientists, values, personal experiences of nature loss, and demographic characteristics were collected. Research shows these factors have been shown to influence environmental sustainability and climate change attitudes, behaviours and policy support^22–25^ (supplementary note 2 details variable rationale, details and measurement). Principal component factor analysis (PCA) identified underlying mental model structures by revealing groups of causal beliefs, and policies and behaviours. Here, mental models are patterns of associations between causal beliefs that structure public support for transformative, policy and behaviour change. So ordinary least squares regression analysis (with robust standard errors) is used to shed light on associations between support for different solutions and causal beliefs, as well as psychological and socio-demographic predictors.

## Results

### Low awareness but high acceptance

The results reveal a striking awareness—acceptance paradox. Only 28% of respondents had heard of the sixth mass extinction event, and merely 16% recognized the term ‘sixth mass extinction’. Yet once provided with a scientific explanation, 93% agreed that the phenomenon is occurring, with 95% attributing it to human activities (see supplementary note 3, Fig. S1–S3).

**Fig. 1:**
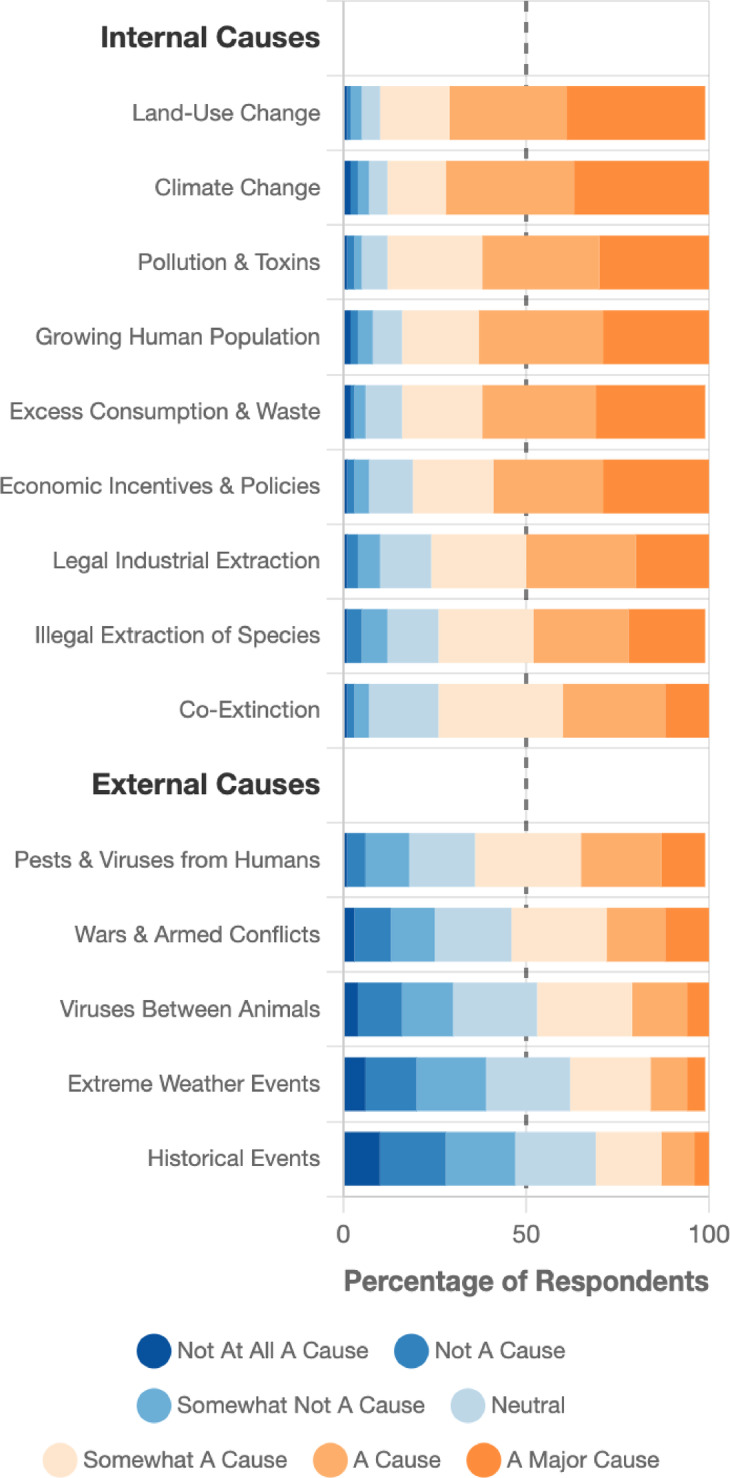
Perceived causes of the sixth mass extinction.

Participants identified land-use change, climate change, and pollution as primary extinction drivers (Fig. 1). PCA revealed two causal belief dimensions: direct human causes (e.g., land-use change, resource extraction, anthropogenic climate change; 33% variance explained) and indirect/distal causes (e.g., animal diseases, historical events like colonialism; 22% variance explained). These components broadly reflect internal and external loci of causality^26^ (i.e., attribution to proximal human factors [internal causes] and distal human and nonhuman factors [external causes]), which is crucial for structuring what causes the SME in a mental model, and therefore how to respond. Items in these clusters were averaged to form two causal belief variables relating to internal and external causal beliefs (supplementary note 4 details PCA results and Table S11 summarises items in each causal belief cluster).

**Fig. 2:**
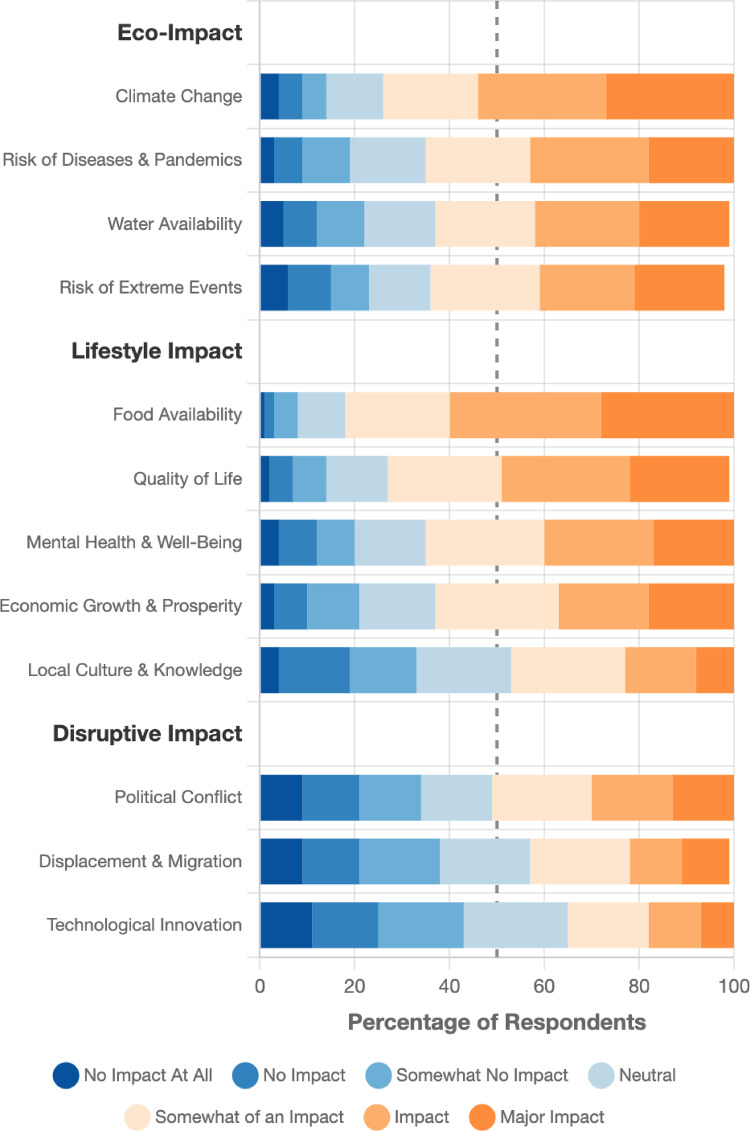
Perceived consequences of the sixth mass extinction.

For consequences, participants reported more adverse impacts on food and water availability, disease risk, and economic prosperity, especially outside the UK (Fig. 2). Three impact clusters emerged: ecological and environmental consequences (e.g., climate change, extreme events; 30% variance), socio-economic and lifestyle impacts (e.g., food availability, economic effects; 19% variance), and disruptive consequences (e.g., conflict, displacement; 19% variance). Items in these clusters were averaged to form three causal belief variables relating to perceived ecological, lifestyle, and disruptive consequence beliefs (supplementary note 4).

### Mental models of transformative change

**Fig. 3:**
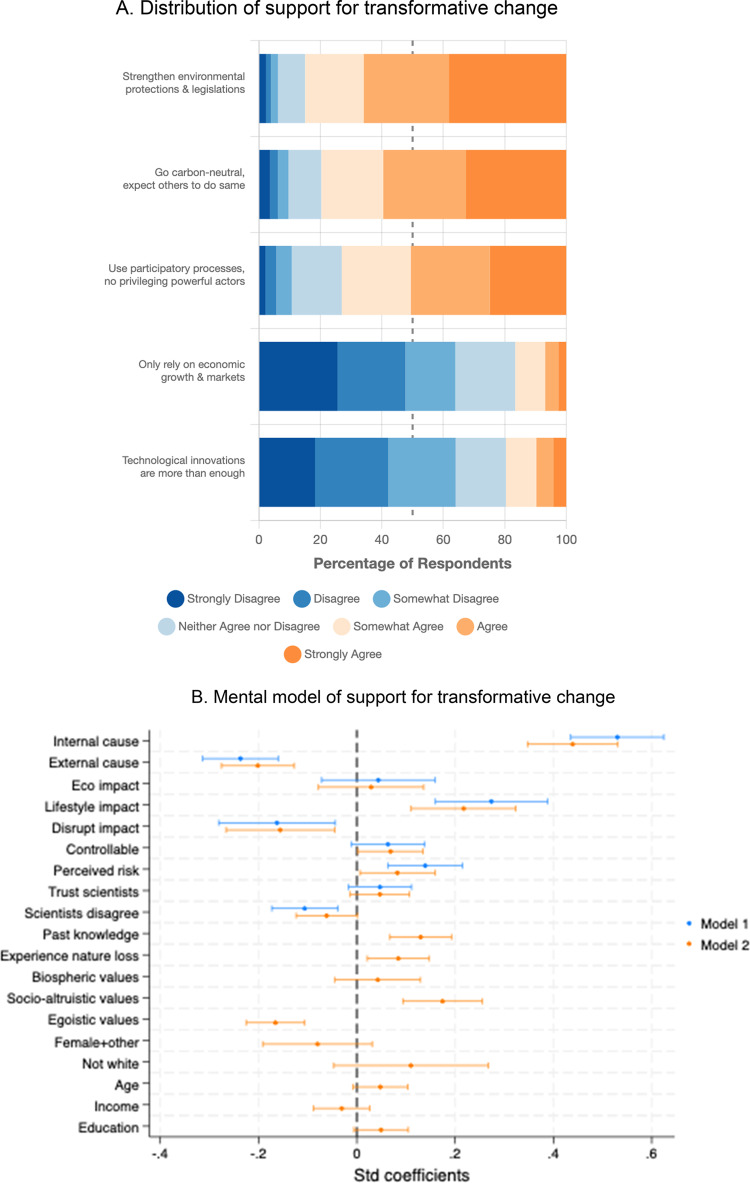
Public support for systems transformations.

Strong support emerged for a transformative approach to change (Fig. 3A), with 85% agreeing that voters, governments, and businesses should strengthen environmental protections, 80% supporting carbon neutrality, and 73% favouring participatory stakeholder processes. Conversely, 64% rejected relying solely on economic growth and technological solutions. The transformative approach to change items were averaged to form a composite support for transformative approach to change variable (Cronbach alpha = 0.659; M = 2.122, SD = 0.998).

Regression analysis revealed that attribution to direct human causes was the strongest predictor of transformative change support (standardized β = 0.44, *p* < 0.001, 95% CI [0.347, 0.531]), while external attributions reduced support (β = − 0.20, *p* < 0.001, 95%CI [− 0.275, − 0.127]; Fig. 3B). Perceived lifestyle impacts positively predicted support (β = 0.22, *p* < 0.001, 95% CI [0.110, 0.324]), while disruptive consequences was negatively associated (β = − 0.16, *p* < 0.01, 95% CI [− 0.265, − 0.044]). Turning to other psychological predictors, socio-altruistic values and experience of nature loss positively predicted support, while egoistic values and perceived disagreement among scientists were negatively associated.

### Distinct policy and behavioural mental models

**Fig. 4:**
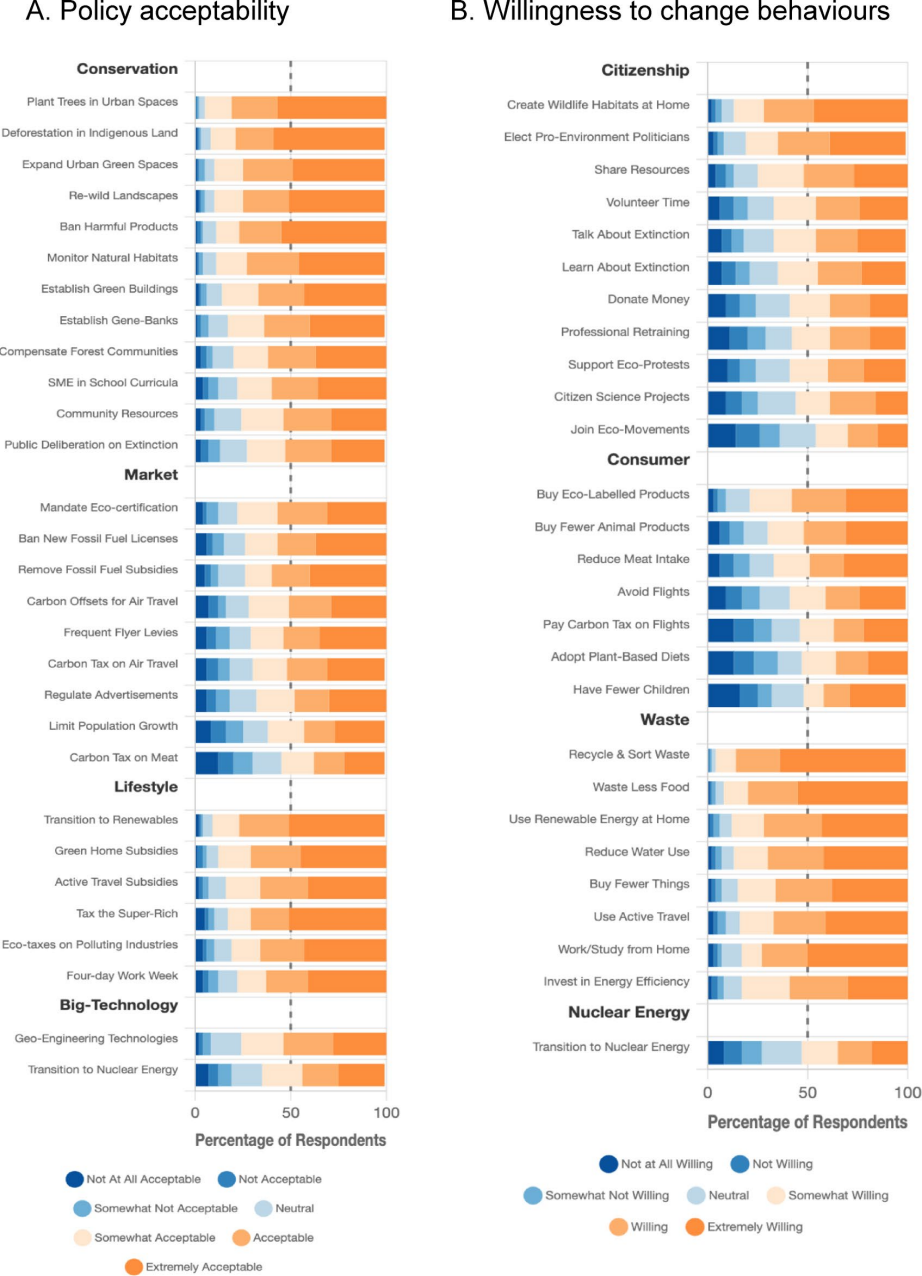
Distribution of support.

All the policies were at least somewhat acceptable to at least 50% of respondents (Fig. 4A). The most popular policies were planting more trees in urban spaces, preventing deforestation in recognised indigenous territories, expanding community urban green spaces, rewilding and managing natural habitats (nearly 90% found them at least somewhat acceptable). Although they were still deemed somewhat acceptable by at least 50% of respondents, the policies with the least acceptance were humanely limiting population growth, regulating commercial advertising, transitioning to nuclear energy and enacting carbon taxes on meat.

For each type of behavioural change except participation in ecological citizen science projects, at least 50% of the respondents were at least somewhat willing to implement the change (Fig. 4B). Respondents were most willing to use renewable energy in their home, reduce water usage, waste less food, recycle and sort waste, work from home, and elect politicians committed to change (over 80% found them at least somewhat acceptable). However, fewer participants were willing to participate in ecological citizen science projects, transition to nuclear energy, reduce meat intake or adopt plant-based diets and have fewer or no children.

PCA identified four policy mental models explaining 63% of variance: Conservation policies (e.g., urban tree planting, habitat protection, eco-education; 24% variance), market regulation (e.g., fossil fuel bans, carbon offsets; 20% variance), socio-economic and lifestyle transitions (e.g., renewable energy, wealth taxes; 14% variance), and big technology interventions (nuclear energy, geoengineering; 6% variance). Similarly, four behavioural models emerged (63% variance): Citizenship actions (e.g., voting, volunteering, education; 25% variance), consumer choices (e.g., eco-products, plant-based diets; 20% variance), waste reduction (e.g., food waste, water conservation; 14% variance), and nuclear energy use (5% variance). Items in each cluster were averaged to form eight composite variables, each corresponding to a policy or behavioural mental model. For the sake of consistency and ease of interpretation1⁹, indices for the perceived effectiveness of policy and behaviour clusters were computed using the same items as for the components themselves, and these items are used as predictors in the regression models discussed below.

### Causal beliefs shape policy and behaviour support

**Fig. 5:**
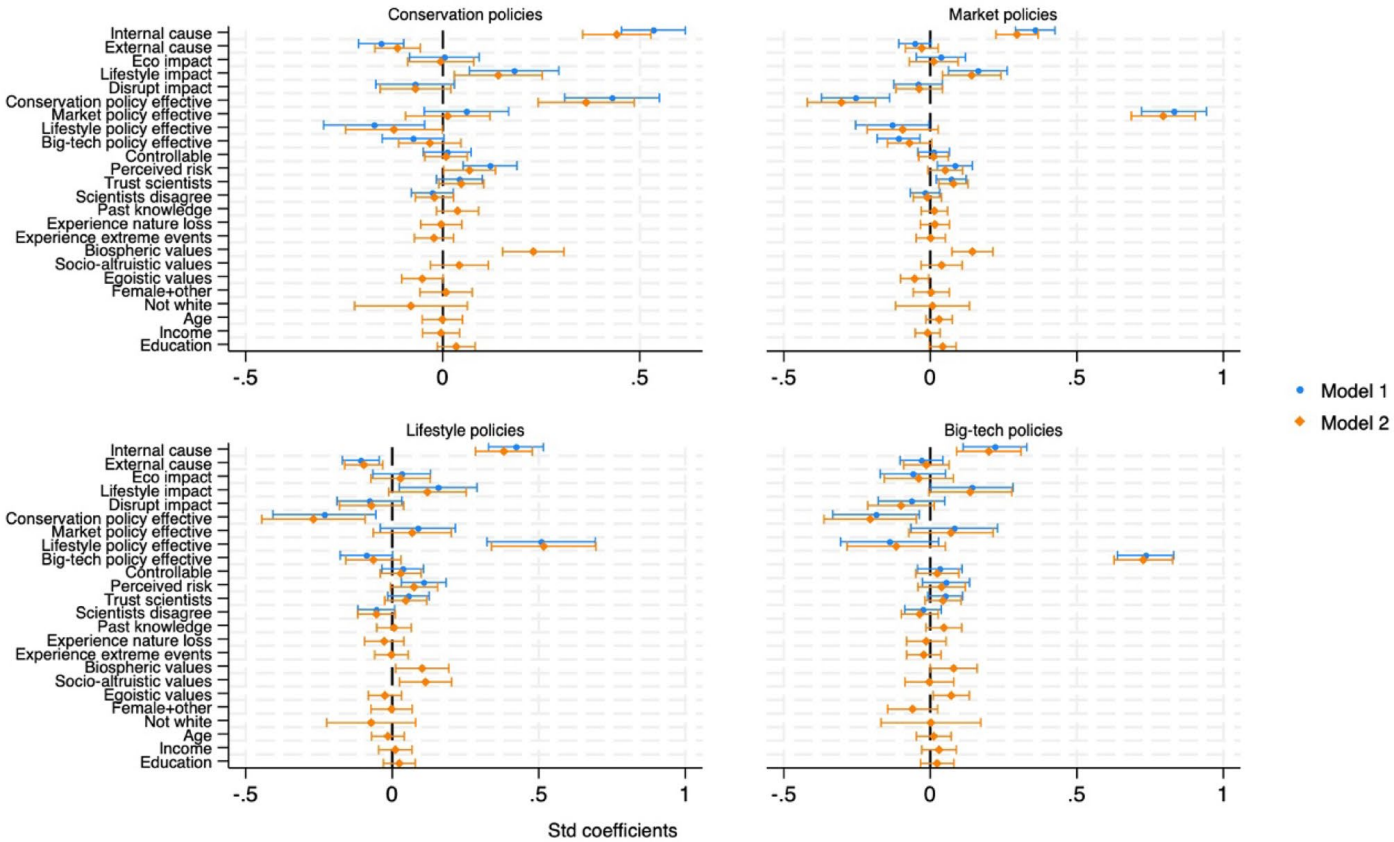
Mental models of policy acceptability.

Attribution to direct human causes consistently predicted support across policy models, with strongest effects for conservation policies (β = 0.44, *p* < 0.001, 95% CI [0.355, 0.528]; Fig. 5). External attributions negatively predicted conservation and lifestyle policy support. Perceived lifestyle impacts enhanced support for conservation, market, and lifestyle policies but not technology interventions. Biospheric values also predicted support in all policy mental models except technology, which was positively associated with egoistic values instead. Notably, perceived effectiveness of conservation policies negatively predicted support for other policy types, suggesting potential trade-offs between approaches. This pattern indicates that different mental models may compete rather than complement each other, with important implications for communication strategies.

**Fig. 6:**
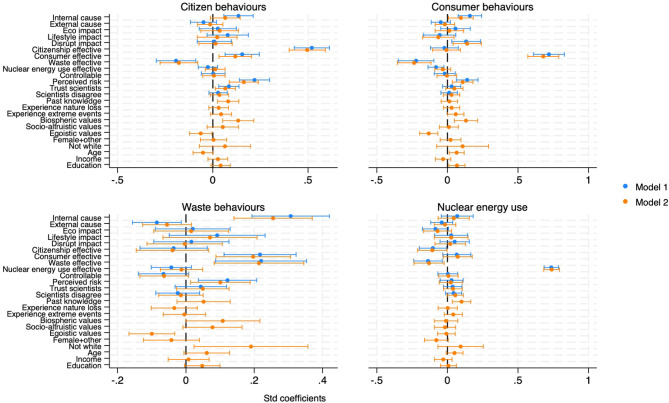
Mental models of willingness to change behaviours.

For willingness to change behaviours, perceived effectiveness dominated as the primary predictor across all models (Fig. 6). Internal causal attributions positively predicted only consumer and waste reduction behaviours. Again, trade-offs emerged, with waste reduction effectiveness negatively associated with support for other behavioural domains. Past knowledge, identification as female or other, and non-white ethnicity were positively associated with citizenship. Income was negatively associated with consumer actions.

## Discussion

The aim of this study was to map public understanding of the SME and support for transformative systems, policy and behaviour change. Currently, evidence on public views is lacking, although such insights are crucial for public communications and policy engagement campaigns. Insights about mental models of support shed light on which changes are deemed acceptable and how psychological factors such as causal beliefs act as barriers or levers to action. The findings reveal both opportunities and challenges for sustainability transformations.

### Implications for sustainability science and policy

The results show low public awareness about the term "the sixth mass extinction" but high acceptance once the term was described. This awareness-acceptance paradox suggests substantial untapped potential for public engagement. Most participants agreed with the need for sustainable and just systems transformations, and the majority supported most policy and behaviour changes. High acceptance and majority support for change may reflect the salience of interlinked phenomena such as climate change which the public already has exposure to. For example, evidence from other nationally representative surveys shows around 86% of British public think climate change is happening and 82% say that the issue is at least “somewhat” important to them personally^27^. More broadly, these results align with recent findings showing a substantial majority is concerned about anthropogenic climate change and support actions the UK^27–31^ (and similar regions such as the EU^32^).

Despite persistent majority support, however, there is a worrying trend of declining support according to recent opinion polls, and the jostling of environment versus other policy priorities^31,33^. Climate and conservation policy is severely lagging and this may be due to several possible reasons. At the individual level, there is widespread “pluralistic ignorance” where both policymakers and the public underestimate the extent of support for action^31,34,35^. Structural and systemic barriers include disinformation and delay campaigns^15,16,25^, media bias in portraying climate issues^36,37^, geopolitical regime shifts and short electoral cycles and type of electoral systems^38,39^, criminalisation of environmental protest^40^, and a disconnect between political narrative versus action^41^.

The findings from this study may be used to enhance support for transformative change by helping address some of the individual-level and structural factors discussed above. For instance, to address individual-level factors such as pluralistic ignorance, public education and awareness campaigns can communicate that the majority of the UK public is concerned about, and supports action to address biodiversity loss and mass extinction, a form consensus messaging^34,35,42^. To counter structural factors like extinction denial and delay campaigns, communicating that there is a scientific consensus that the crises is anthropogenic with severe consequences for human wellbeing could also be helpful. This study finds that emphasizing direct human causes of biodiversity loss, and socio-economic and lifestyle consequences, predicts support for transformative action. This finding is in alignment with lessons from climate and environmental communications research which finds that communicating the scientific consensus about human causes^23,43,44^, issue relevance^45,46^, severity of impacts, and the benefits of effective (and just) policies using trusted advocates like scientists^47,48^ can increase policy support across contexts.

The identification of distinct mental models highlights the potential for tailored engagement strategies. Effective communication should recognize that different population segments may be more receptive to conservation-focused, market-based, lifestyle-oriented, or technology framings. This is similar to findings from Bostrom et al.^20^ who find three climate mitigation policy mental models, namely “green” (similar to conservation and lifestyle policies), “carbon” (similar to market regulation) and “engineering” (similar to big technology). Both this study, and Bostrom et al.^20^, also find the evidence for trade-offs between mental models, where for example, people who perceive technology interventions are more effective are less likely to support conservation or carbon reduction policies. These perceived trade-offs raise concerns about unintended consequences of narrow, targeted communication strategies. If emphasizing conservation policy effectiveness reduces support for market or lifestyle approaches, campaigns may inadvertently limit the comprehensive response needed for transformative change, which requires system-wide actions across all domains. Future research should investigate these trade-offs more thoroughly and explore whether co-benefits framing can mitigate unintended effects where appropriate^49^.

### Pathways for transformative sustainability action

These results suggest three key pathways for advancing sustainability transformations:

#### Framing in science communication

The low awareness-high acceptance pattern indicates significant potential for scaling biodiversity crisis communication from specialist contexts so that SME enters mainstream discourse. Direct human causes, action effectiveness, and scientific consensus framings could increase engagement with support for transformative change, policy acceptability and willingness to change behaviours.

#### Values-based public engagement

The positive association between socio-altruistic values and transformative support suggests that sustainability messaging could emphasise collective benefits and shared responsibility, rather than individual costs or benefits, or technological fixes alone. In line with studies suggesting that values are crucial determinants of climate and environmental policy support^46,50^, cultivating and strengthening socio-altruistic values through education and civic programmes will also be valuable.

#### Integrated solution framing

Whilst distinct mental models exist, the broad support for various approaches (> 50% for most policies and behaviours) indicates potential for comprehensive sustainability strategies that combine conservation, market, lifestyle, and technological elements. Perceived effectiveness is especially key to communicate, especially for behaviour change campaigns^46^. Perceived trade-offs could be explicitly addressed by making co-benefits explicit, and prioritizing solutions where there are co-benefits to be leveraged^51,52^. Highlighting the majority support for policy, behaviour, and transformative change may also help tackle pluralistic ignorance about the public willingness to act, which has been found as a barrier to support in other research^53,54^.

### Study limitations and future directions

Several limitations warrant consideration and should be addressed in future research. The UK sample, while nationally representative, may not generalize to other cultural contexts where environmental attitudes, political systems, and economic conditions differ significantly. The higher education levels in our sample compared to national averages may also limit generalisability in the UK itself.

Additionally, stated support for policies and behaviours may not translate directly into actual changes on the ground due to intention-behaviour gaps^55^ and structural barriers preventing action^56^. For instance, carbon-lock of travel infrastructures, or insufficient bus services may hinder individuals in moving towards greener or public transport, thus mitigating the impact of any framing or communication approaches^56,57^. Future research should examine how mental models relate to real-world choices to take sustainability and policy actions, and the role of structural barriers in mitigating action.

This study aimed to understand public awareness of and different mental models for supporting action against biodiversity loss and mass extinction. However, support for actual policies may hinge on policy design features, such as who bears the cost of the policy and how it is rolled out, apart from whether it is effective. Factors such as perceived fairness will therefore be an important consideration^29,35,45,58^. Regressive policies are especially unpopular as starkly illustrated by the *Gilets Jaunes* protests in France against carbon taxes^59^. Thus, to understand whether specific policy proposals will be supported on the ground, careful policy design testing attributes of different policies will be required. Directly involving people in the co-development of biodiversity policies, for example through citizen-led forums like deliberative assemblies^60,61^, may be useful to disseminate the science of biodiversity loss and to move from public understanding to public engagement^62^. In this regard the framing approaches suggested in this study can be a valuable complement to carefully designed policy and compatible with deliberative approaches, but not a substitute.

Cross-cultural replication is particularly important given varying exposure to biodiversity loss, environmental governance systems and cultural values globally. Studies in developing countries, where biodiversity loss impacts may be more immediate but resources for action more constrained, could reveal different mental model structures^54^a. In addition, psychological factors such as perceived fairness should also be explored in relation to policy and behaviour change acceptability^63^.

## Methods

### Survey development and implementation

The study was approved by the London School of Economics and Political Science ethics committee and was performed in accordance with relevant guidelines and regulations. It was pre-registered (https://osf.io/ywb9z/?view_only=cf975b2d66df426d992912892217cd21). The study design followed a mental models approach21 (see supplementary note 1 for explanation of this approach and minor deviation from the pre-registration). Normative research (on what the public should understand) included literature review and expert interviews (n = 10 conservation scientists). This phase of the research was primarily focused on narrowing down the description of the topic and the most relevant items for causes, consequences, behaviour, policy and psychological questions. Descriptive research (on the public’s actual understanding) involved focus groups (n = 32 graduate students) and pilot surveys (n = 79 UK residents) to develop the final instrument and to ensure clarity and comprehension of the questions, and survey timing and experience.

### Participants and data collection

750 UK residents aged 18 + were recruited through Prolific Academic (July 4 and 5, 2022) with demographic quotas. Informed consent was obtained from all participants. No identifying information was collected from any participant. The survey was hosted on Qualtrics. Quality controls for response quality included CAPTCHA verification, attention checks, minimum 8-min completion time and 90% platform approval rating requirement. The final sample was n = 739 after exclusions.

### Measures

The survey assessed: (1) awareness and beliefs about sixth mass extinction; (2) perceived causes and consequences using 7-point scales; (3) support for transformative change, policies, and behaviours using 7-point scales; (4) psychological factors including values, risk perception, and scientific trust; (5) socio-demographics and personal experiences (see supplementary note 2 for details of variables measurement including which pre-validated scales were used).

### Statistical analysis

We conducted PCA with varimax rotation (Kaiser criterion) to identify causal beliefs and policy and behaviour mental model dimensions. Conducting PCA on the items measuring causal beliefs and support for policy and behaviour change will show a subset of core dimensions or components identified as critical for transformative change. The items grouped into each category are based on factor loadings (barring a few instances where the author’s judgment came into play, for example when it made theoretical sense to group a particular item into a component given the factor loading and the rest of the items in that component; additional analyses revealed no substantive difference in the results in these cases). The values of items belonging to the component are averaged to form composite variables capturing different categories of policy and behaviour change, which forms the basis of a policy or behaviour mental model (see supplementary note 4 for explanation of the rationale behind PCA and results). In this study, therefore, mental models are patterns of associations between causal beliefs that structure public support for transformative, policy and behaviour change. Ordinary least squares regression with standardised variables and robust standard errors examined relationships between causal beliefs and solution support (Supplementary note 5 for rationale and details of the results). To obtain mental models of solutions, support for systems transformations and different clusters of policies and behaviours were regressed on causal beliefs, psychological and socio-demographic factors in each regression model. The variance inflation factor was less than the conventional standard of 10 for all the estimated models, suggesting that there was no multicollinearity. All analyses were conducted in Stata 16.

## Supplementary Information

Below is the link to the electronic supplementary material.

Supplementary Material 1

## Data Availability

All data and analysis code is available at the Open Science Foundation: https://osf.io/cs6a2/overview?view_only=d5af6fd2aea548498f94589df3c9b0cb.
