# Supplementary material for: Mental models of the sixth mass extinction reveal pathways for transformative sustainability action
Source: Sci Rep. 2026 Feb 20;16:10004. doi: 10.1038/s41598-026-40100-w (PMC13022189; doi:10.1038/s41598-026-40100-w)
Supplement: Supplementary file 1 — Supplementary Material 1 [file 41598_2026_40100_MOESM1_ESM.docx]

# Electronic Supplementary Materials

# Supplementary note 1: Mental models approach used in this study

This is a specific methodology used in science communication that stems from environmental psychology research and has two main phases^1^:

1. **Normative Research Phase** ("What Should People Know?"): This phase asks: According to experts, what do people need to know to make informed decisions about this topic? In this study, the researcher interviewed 10 conservation biology and behavioural science experts. These experts represented diverse backgrounds across regions, genders, sectors, and seniority levels. The goal was to identify what scientific knowledge is most crucial for public understanding of the sixth mass extinction. Secondly, a review of scientific, peer-reviewed literature, policy documents and science communication materials were conducted (see supplementary note 2). This helped identify key concepts, causes, consequences, and solutions that experts consider important.

2. **Descriptive Research Phase** ("What Do People Actually Know?"): This phase asks: What do people currently know, what misconceptions do they have, and what are their preferences. For this phase, focus Groups (n=32) were conducted with graduate students studying behavioural science. These discussions revealed how people naturally think and talk about biodiversity loss, and helped identify common misconceptions, knowledge gaps, and language preferences. In addition, pilot surveys (n=50 and n=29) with British public were used to test different question formats and identified prevalent beliefs.

This methodology is valuable because it:

- Mitigates researcher bias: Instead of researchers deciding what's important, experts define the knowledge needs
- Grounds survey questions in actual public thinking by ensuring questions reflect how people conceptualize the topic
- Provides comprehensive coverage of the sustainability issues arising in the context of SME by combining expert knowledge with public perspectives
- Enables both general and targeted communication by identifying specific knowledge gaps through understanding both expert-defined needs and public knowledge.

The insights from both phases were used to create the final survey that forms the basis of the study. This survey included:

- Questions testing awareness of terms experts deemed important
- Items measuring beliefs about causes and consequences identified in both expert interviews and public discussions
- Policy and behaviour options that emerged from both expert recommendations and public preferences

Process: This normative research phase was primarily focused on narrowing down the description of the topic, items for causes, consequences, behaviour and policy questions, and psychological factors. This was done by triangulating the evidence from the literature reviews to arrive at a longlist of items, and then and using the expert interviews to arrive at a shortlist. Variations in the domain of the behaviour (e.g. education, protest and collective action, transport, food etc.), apart from cost and benefit from taking action were considered. Descriptive research (on the public's actual understanding) involved focus groups (n=32 graduate students) and pilot surveys (n=79 UK residents) to develop the final instrument and to ensure clarity and comprehension of the question text and flow, and survey timing and experience.

Sample: Participants were recruited through [Prolific Academic](https://www.prolific.com/) using demographic quotas to ensure the sample matched UK population on key characteristics. To ensure quality, only participants with 90% approval ratings (indicating reliable participation in previous studies) were allowed to participate. In addition, the study excluded anyone who had participated in the pilot studies. Several other attention checks and quality measures were also used (i.e., CAPTCHA verification to weed out any survey bots, attention check questions embedded in the survey and a minimum 8-minute completion time). This methodology helped ensure that the final survey captured both expert priorities and public thinking patterns, while maintaining scientific rigor through pre-registration and representative sampling. It's designed to produce actionable insights for science communication and policy engagement.

Pre-registration and data availability statements: The study was pre-registered on the Open Science Foundation: <https://osf.io/ywb9z/?view_only=cf975b2d66df426d992912892217cd21>, where the entire survey including the consent information, main outcomes and socio-demographic variables, was specified. The analyses were data-dependent and exploratory. The only deviation from the pre-registration was that perceived responsibility and collective efficacy was not included in the regressions because they were highly correlated with perceived risk and concern (r>0.5).

The data and analysis code will be uploaded on OSF project webpage after the article is published. It is available for peer review at this OSF link: <https://osf.io/cs6a2/?view_only=6d4cd9360b664742940da84e1dc51a3f>.

# Supplementary note 2: Survey Questions and Variables

Table S 1: Survey questions, reliability coefficients and source

| Construct | Items and Response scale | Cronbach alpha^ | Bibliographic source* |
| --- | --- | --- | --- |
| Awareness | Have you heard about the term the sixth mass extinction before?  Have you heard about the term “ecosystem” before?  Have you heard about the term “biodiversity” before?  Response:  o Definitely not (1)  o Probably not (2)  o Maybe (3)  o Probably yes (4)  o Definitely yes (5) | 0.7346 | ^2^ |
| Human cause | Now we will consider the causes of the sixth mass extinction. Assuming that mass extinction is happening, do you think that it is...   - Caused mostly by human activities (1) - Caused mostly by natural changes in the environment or natural factors (2) - Caused in equal part by separate human activities and natural factors (3) - Caused mostly by human activities, which then interact with natural factors (4) - Caused mostly by natural factors, which then interact with human activities (5) - It is not happening (6) - Don't know (7) | NA | ^2^ |
| Causes | To what extent are each of the following items responsible for causing today's mass extinction?  Your answers can range from 1 (= Not at all a cause) to 7 (= Major cause).   - Land-use change (e.g. forests converted to farms & cities) - Holes in the ozone layer - Illegal extraction of animals and plants (e.g. poaching, illegal trade) - Legal industrial extraction of animals and plants (e.g. fishing, logging) - Climate change (e.g. hotter temperatures) - Harmful pests and viruses bought in by humans (e.g. trade, travel or tourism) - Growing human population - Excess consumption and wasteful lifestyles - Pollution and toxins from producing goods and services (e.g. electronics and energy) - Harmful economic incentives and policies leading unsustainable practices (e.g. fossil fuel subsidies) - Wars and armed conflicts - Historical events (e.g. colonialism) - Extreme events (e.g. volcanoes) - Viruses passed on between animals - Co-extinctions (loss of one species causes loss of others)   Response:  Not at all a cause 1 (1)  2 (2)  3 (3)  4 (4)  5 (5)  6 (6)  Major cause 7 (7) | (see factor analysis; Table 1 in main text) | ^3,4^ |
| Consequence | How much do you think that mass extinction will affect the following issues in the UK to the best of your knowledge?   - Food availability (e.g. soil fertility, pollination services, agricultural pests) - Water availability (e.g. droughts) - Local culture and knowledge (e.g. animals and nature) - Mental health and well-being - Economic growth & prosperity - Displacement and migration of UK residents - Risk of diseases and pandemics - Quality of life (e.g. living standards, consumption) - Political conflict (e.g. wars) - Technological innovation - Risk of extreme events (e.g. floods, hurricanes) - Climate change (e.g. global warming)   Response:  No impact at all (1)  2 (2)  3 (3)  4 (4)  5 (5)  6 (6)  A major impact (7)  Note: this question was asked once in the context of the UK and once outside the UK. | (see factor analysis; Table 1 in main text) | ^4,5^ |
| Perceived risk and concern | Now we would like to know more about how you feel about the sixth mass extinction.   - How much does the idea of mass extinction fill you with dread, if at all?   - Response using slider from Not at all concerned (1) to Extremely concerned (7) - How concerned are you about mass extinction?   - Response using slider from Not at all concerned (1) to Extremely concerned (7) - How serious a threat is mass extinction to you personally?   - Response using slider from Not at all a serious threat (1) to Extremely serious threat (7) | 0.8606 | ^3,6^ |
| Controllability | Assuming that mass extinction is happening, to what extent do you think that it can be controlled by humanity?  Response using slider from Not at all controllable (1) to Extremely controllable (7) | NA | ^3^ |
| Policy acceptability | We can also support and implement several policies to stop, reduce or delay mass extinction. How acceptable to you, are the following policy options in the UK? Your answers can range from 1 (= Not at all acceptable) to 7 (= Extremely acceptable).   - Ban new fossil fuel licenses (e.g., oil, coal) - Remove fossil fuel subsidies - Transition energy systems to renewables - Transition energy systems to nuclear energy - Re-wild natural and agricultural landscapes - Stop industrial harvesting (e.g. trawling) in protected areas - Mandate eco-certification for companies and eco-labels for consumers - Prevent deforestation in recognised Indigenous territories - Enforce, manage and monitor wild species, natural habitats, and landscapes - Regulate commercial advertisement persuading people to buy more stuff - Expand community urban green spaces (e.g., neighbourhood gardens, urban agriculture) - Mandate carbon offsets for air travel - Carbon taxes for air travel - Four-day work week (longer hours/day+no pay reduction) - Frequent flyer and business class flight levies - Green home (e.g. insulation, retrofitting) & electric vehicle subsidies - Active travel subsidies (e.g. bike schemes) - Carbon tax on meat - Invest in shared community-based resources (e.g. micro-grids) - Include sixth mass extinction in school and university curricula - Public deliberation about extinction and biodiversity loss to gauge policy preferences - Wealth and carbon taxes on the super-rich - Monetary compensation to indigenous and local communities protecting tropical forests - Higher eco-taxes on polluting and extractive industries (e.g. multinational fossil fuel and agricultural companies) - Limit population growth humanely (e.g. encourage contraception use) - Plant more trees in urban and degraded spaces - Adopt geo-engineering technologies - Establish green buildings (e.g. planting on walls & roofs to attract animals) - Establish animal and plant gene-banks to preserve genetic diversity - Ban harmful products and pesticides (e.g. neonicontinoids harms bees, plastic pollutants harm whales)   Response:  Not at all acceptable 1 (1)  2 (2)  3 (3)  4 (4)  5 (5)  6 (6)  Extremely acceptable 7 (7) | (see factor analysis; Table 1 in main text) | ^3,4,7–9^ |
| Willingness to change behaviours | There are many actions we can take in our everyday lives to stop, reduce or delay mass extinction. These actions can have more or less negative impacts on ecosystems and biodiversity. How willing are you to take the following actions? Your answers can range from 1 (= Not at all willing) to 7 (= Extremely willing).   - Invest in energy and water efficiency devices - Use renewable energy in your home - Reduce water use (e.g. shorter showers) - Transition to nuclear energy - Reduce intake of meat and animal proteins - Waste less food (e.g. reusing leftovers, composting) - Recycle and sort waste - Adopt plant-based diets - Work and study from home - Use active travel (e.g. walking and cycling) - Avoid flights - Pay for carbon offsets and carbon taxes when flying - Buy fewer things (e.g. shop less, reuse clothes, avoid plastic) - Have no or fewer children - Buy fewer animal products (e.g. less leather, more plant-based items) - Buy sustainably-sourced and eco-labelled products - Elect politicians committed to halting extinction and climate change - Share resources with others (e.g. micro-grids, car-sharing) - Support eco-protests and green petitions - Participate in eco-movements (e.g. attending meetings, sharing on social media) - Retrain professionally to enact sustainability at home and work (e.g. green skills and courses) - Spend time getting to learn about extinction debates and issues (e.g. radio, news) - Talk to friends, family and colleagues about extinction - Provide habitats for wildlife in gardens/balconies - Volunteer time to restore nature (e.g. planting trees, community gardens, wildlife rescues) - Donate money to conservation projects & wild animals - Participate in ecological citizen science projects   Response:  Not at all willing 1 (1)  2 (2)  3 (3)  4 (4)  5 (5)  6 (6)  Extremely willing 7 (7) | (see factor analysis; Table 1 in main text) | ^3,4,7–9^ |
| Science views | Trust in science  In general, how much do you trust scientists?  Response:  O Don't trust at all (1)  o Don't trust a lot (2)  o Trust somewhat (3)  o Trust completely (4)  In your opinion, how much disagreement is there between scientists that this mass extinction is happening?  Response:  o None at all (1)  o A little (2)  o A moderate amount (3)  o A lot (4)  o A great deal (5)  o Don't know (99)  Perceived disagreement amongst scientists  In your opinion, how much disagreement is there between scientists that this mass extinction is caused by humans?  Response:  o None at all (1)  o A little (2)  o A moderate amount (3)  o A lot (4)  o A great deal (5)  o Don't know (99) | NA | ^10,11^ |
| Past experience | Considering roughly the last 5 years, how often (in total) have you personally experienced any type of extreme weather event in your local UK area? (e.g., severe heat waves, droughts, storms, hurricanes, flooding etc.).  Response:  o Never (1)  o Once (2)  o Twice (3)  o Three or more (4)  o Can't remember (99)  Considering roughly the last 5 years, have you personally experienced a decrease in local nature (e.g. growth of insects, birds or animals, and/or restoration of previously polluted parks, rivers, beaches etc.) your local UK area?  Response:  o Definitely not (1)  o Probably not (2)  o Might or might not (3)  o Probably yes (4)  o Definitely yes (5) | NA | ^6^ |

Note: Cause, Impact, Policy and Behaviour items were elicited to scientific peer reviewed literature, IPBES and IPCC reports, as well as science communication efforts such as [NHM](https://www.nhm.ac.uk/discover/what-is-mass-extinction-and-are-we-facing-a-sixth-one.html) and [Extinction: The Facts](https://www.bbc.co.uk/iplayer/episode/m000mn4n/extinction-the-facts), apart from being cross-checked with expert interviews. They reflect variations along several dimensions including impact, domain, personal cost and effort, environmental impact, popularity. Other variables were collected in the dataset which were not used in the analysis (see pre-registration). *See reference list.^ Cronbach's alpha is a measure of internal consistency, indicating how closely related a set of items are as a group. A higher Cronbach's alpha (closer to 1) suggests greater internal consistency, implying that the items are indeed measuring the same construct. Don’t know responses are excluded.

**Mass extinction explanation and question**

Mass extinction refers to the idea that the variety and number of animal and plant species are vanishing much faster than they are replaced. Before humans, there have been five mass extinction events where 76% of species were lost (e.g., the dinosaur extinction).

Mass extinction is different from the occasional species going extinct. Some animals and plants can go extinct over time. But slowly, over a period of several million years, their role in a given ecological system or ecosystem (a natural environment with plants and animals living and interacting within it) can be balanced. This happens through the creation of new (and often very different) species or a change in local populations of existing ones.

In this way, earth's “normal” extinction rate is often thought to be somewhere between 0.1 and 1 species per 10,000 species per 100 years. This normal rate is also known as the pre-human or background rate of extinction.

Now, scientists think that we are in the midst of the sixth mass extinction event– the current extinction rate is between 100 and 1,000 times higher than the pre-human rate of extinction.

Now, local populations of several species are fast shrinking and an estimated 1 million animal and plant species currently face extinction. This means an irreversible loss in biodiversity (the variety of species found in an ecosystem) and life in nature as we know it, even during our lifetimes.

We refer to the sixth mass extinction as “mass extinction” in this survey. Do you think that mass extinction is happening?

*Response scale:* Definitely not happening (1) Probably not happening (2) Probably happening (3) Most likely happening (4) Definitely happening (5) Don't know (6)

*Sources:*

- Explanation of mass extinction from peer-reviewed articles (e.g. Ceballos et al., 2020; Cowie et al., 2022) and science communication (e.g. [NHM](https://www.nhm.ac.uk/discover/what-is-mass-extinction-and-are-we-facing-a-sixth-one.html) and [Extinction: The Facts](https://www.bbc.co.uk/iplayer/episode/m000mn4n/extinction-the-facts)).
- Definition of biodiversity and ecosystem from [BBC GCSE bitesize](https://www.bbc.co.uk/bitesize/guides/zwh9j6f/revision/1#:~:text=An%20ecosystem%20is%20a%20natural,living%20components%20of%20the%20ecosystem.).
- Overall framing and response scales ^2^

**Transformative approach to change explanation and question**

We will now think of different ways to stop it, reduce it or delay mass extinction. We would first like to know whether you think that we should adopt a transformative approach to address mass extinction.

A transformative approach means doing things very differently— not just a little more or less of something we’re already doing.

Truly transformative change often starts small, but becomes sweeping. It includes changes to individual decisions that can start new ways of acting in society, to significant political, economic and legal changes that organisations and countries can make.

Your answers can range from 1 (= Strongly disagree) to 7 (= Strongly agree). There are no right or wrong answers, we only care about what you think.

To address the sixth mass extinction...

- Voters, governments and businesses should strengthen local & global environmental protections & legislations.
- We should use participatory processes involving all major stakeholders, without privileging powerful actors & vested interests.
- We need to go carbon-neutral, and expect others and businesses to do the
- same.
- We need to only rely on economic growth and markets. (R)
- Technological innovations from businesses are more than enough. (R)

*Response scale:* Strongly Disagree (1) to Strongly Agree (7)

Strongly disagree 1 (1)

2 (2)

3 (3)

4 (4)

5 (5)

6 (6)

Strongly agree 7 (7)

*Source:* Adapted from ^14,15^

References:

1. Wong-Parodi, G. & Bruine De Bruin, W. Informing Public Perceptions About Climate Change: A ‘Mental Models’ Approach. *Sci. Eng. Ethics* **23**, 1369–1386 (2017).

2. Leiserowitz, A. *et al.* *Climate Change in the American Mind*. (2021).

3. Bostrom, A. *et al.* Causal thinking and support for climate change policies: International survey findings. *Glob. Environ. Change* **22**, 210–222 (2012).

4. Díaz, S. *et al.* Pervasive human-driven decline of life on Earth points to the need for transformative change. *Science* **366**, eaax3100 (2019).

5. Díaz, S. *et al.* The IPBES Conceptual Framework — connecting nature and people. *Curr. Opin. Environ. Sustain.* **14**, 1–16 (2015).

6. van der Linden, S. The social-psychological determinants of climate change risk perceptions: Towards a comprehensive model. *J. Environ. Psychol.* **41**, 112–124 (2015).

7. Creutzig, F. *et al.* Demand-side solutions to climate change mitigation consistent with high levels of well-being. *Nat. Clim. Change* **12**, 36–46 (2022).

8. Drews, S. & Van den Bergh, J. C. What explains public support for climate policies? A review of empirical and experimental studies. *Clim. Policy* **16**, 855–876 (2016).

9. Sabherwal, A. & Shreedhar, G. Stories of intentional action mobilise climate policy support and action intentions. *Sci. Rep.* **12**, 1179 (2022).

10. Algan, Y., Cohen, D., Davoine, E., Foucault, M. & Stantcheva, S. Trust in scientists in times of pandemic: Panel evidence from 12 countries. *Proc. Natl. Acad. Sci.* **118**, e2108576118 (2021).

11. Ding, D., Maibach, E. W., Zhao, X., Roser-Renouf, C. & Leiserowitz, A. Support for climate policy and societal action are linked to perceptions about scientific agreement. *Nat. Clim. Change* **1**, 462–466 (2011).

12. Ceballos, G., Ehrlich, P. R. & Raven, P. H. Vertebrates on the brink as indicators of biological annihilation and the sixth mass extinction. *Proc. Natl. Acad. Sci.* **117**, 13596–13602 (2020).

13. Cowie, R. H., Bouchet, P. & Fontaine, B. The Sixth Mass Extinction: fact, fiction or speculation? *Biol. Rev.* **97**, 640–663 (2022).

14. Chan, K. What Is Transformative Change, and How Do We Achieve It? *IPBES secretariat* https://ipbes.net/guest-blog-kai-chan-transformative-change (2019).

15. Díaz, S. *et al.* Pervasive human-driven decline of life on Earth points to the need for transformative change. *Science* **366**, (2019).

# Supplementary note 3: Sample descriptive characteristics

Table S 2: Sample descriptive characteristics

| Variable | Obs | Mean | Std. dev. | Min | Max |
| --- | --- | --- | --- | --- | --- |
| Controllability | 739 | 4.744 | 1.320 | 1 | 7 |
| Perceived risk | 739 | 4.496 | 1.544 | 1 | 7 |
| Trust in scientists | 739 | 3.175 | 0.568 | 1 | 4 |
| Perceived disagreement amongst scientists* | 602 | 2.696 | 0.939 | 1 | 5 |
| Past knowledge | 739 | 3.469 | 0.899 | 1 | 5 |
| Past experience of nature decline | 739 | 3.307 | 1.152 | 1 | 5 |
| Female | 739 | 0.521 | 0.500 | 0 | 1 |
| Not white ethnicity | 739 | 0.130 | 0.336 | 0 | 1 |
| Age | 739 | 45.677 | 15.885 | 9 | 90 |
| Education | 739 | 3.626 | 1.090 | 1 | 6 |
| Income | 739 | 4.403 | 2.009 | 1 | 8 |

***** Participants who stated “I don’t know” were omitted.

**Note on representativeness of the sample:**

- Age: The average age was 45.68 years (40.7 years in the U.K. population in mid-2022 =)^1^.
- Gender: Around 48% of respondents self-identified as male and 52% as female (50.98% females and 49.02% males in the U.K. population in mid-2022)^1^.
- Ethnicity: 87% self-identified as white (around 87.4% in the U.K. population)^2^.
- Income: Most respondents reported an annual income between £32,000 and £48,000 (U.K. median income = £31,400)^2^.
- Education status: 79% received at least an undergraduate degree or higher (U.K. Office for National Statistics reports 39% of people in the U.K. ages 15-64 report having received higher education)^3^.
- Note: all socio-demographic and other questions can be found in the survey via the pre-registration link: <https://osf.io/ywb9z/?view_only=cf975b2d66df426d992912892217cd21>

1. Office for National Statistics. (2021). Population estimates for the UK, England, Wales, Scotland, and Northern Ireland: mid-2022. https://www.ons.gov.uk/peoplepopulationandcommunity/populationandmigration/populationestimates/bulletins/annualmidyearpopulationestimates/mid2022.

2. Office for National Statistics. (2020). Population of England and Wales. https://www.ethnicity-facts-figures.service.gov.uk/uk-population-by-ethnicity/national-and-region­al-populations/population-of-england-and-wales/latest.

2. Office for National Statistics. (2022). Average household income: U.K. https://www.ons.gov.uk/peoplepopulationandcommunity/personalandhouseholdfinances/incomean­dwealth/bulletins/householddisposableincomeandinequality/financialyearending2021.

3. Office for National Statistics (HESA). (2022). Higher Education Student Statistics: U.K., 2020/21. https://www.hesa.ac.uk/news/25-01-2022/sb262-higher-education-stu­dent-statistics

Figure S 1: Figure S1: Distribution of participant awareness levels of the sixth mass extinction (SME)


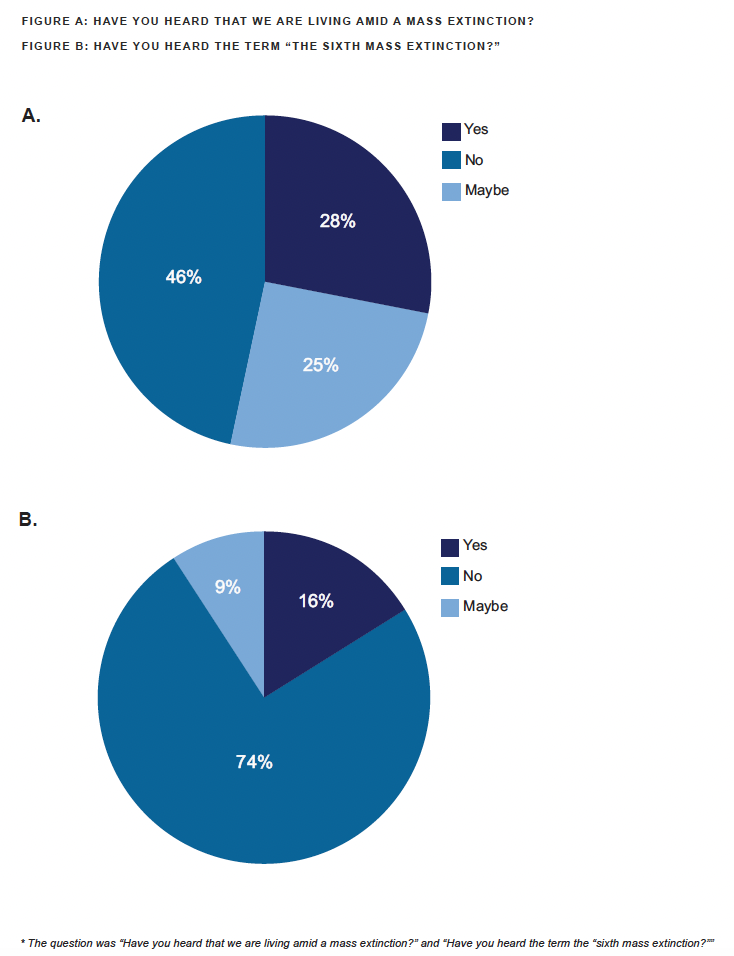


Figure S 2: Is the SME Happening


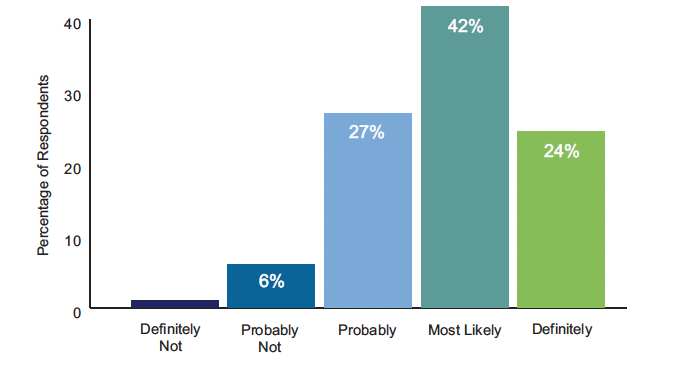


Figure S 3: Main causes of SME

**
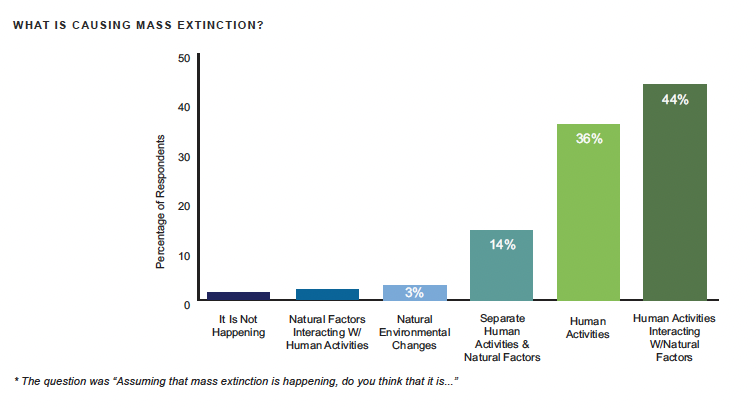
**

Figure S 4: Perceived consequences outside the UK


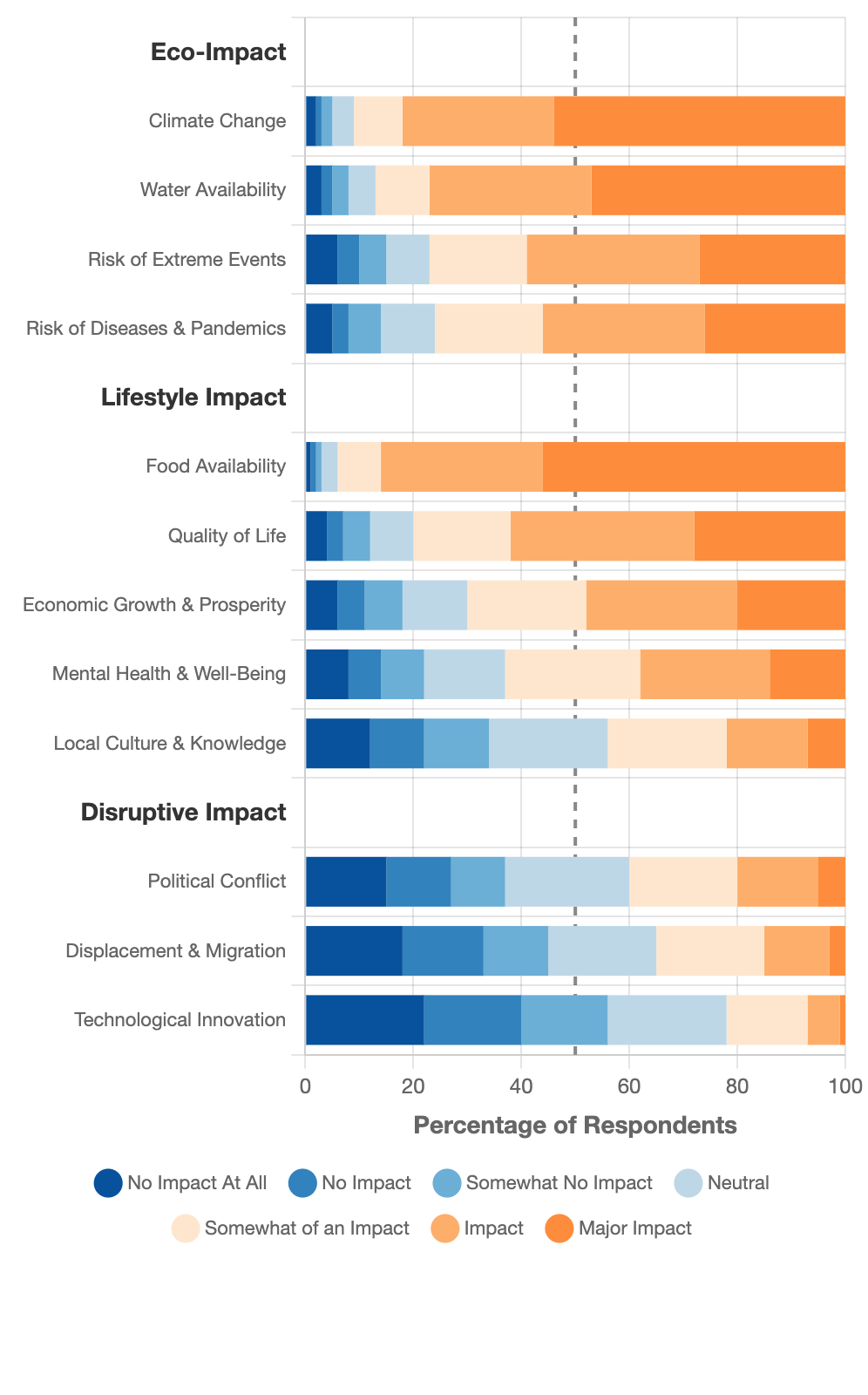


# Supplementary note 4: Principal Components Factor Analysis

Principal Components Factor (PCA) analyses were conducted to obtain reduced number of variables for the sixth mass extinction (SME) causes and consequences beliefs, and support for policy and behaviour change, and understand the structure of the data more broadly. Specifically, in this study, PCA was used to address the following questions:

- What are the clusters of support for policies and behaviours that structure public willingness to address SME through action?

Conducting PCA on the items measuring support for policy and behaviour change, will show a subset of core dimensions or components which are different categories of policy and behaviour outcomes identified as critical for transformative change to tackle the SME. The items in each category are averaged to form composite variables capturing different categories of policy and behaviour change, which forms the basis of a policy or behaviour mental model. Associations between these composite outcome variables and different causal beliefs will be explored in the regression analyses that follows in the next section.

- What are the clusters of causal beliefs regarding the perceived causes and consequences of the SME that structure public understanding of biodiversity loss and extinction?

Conducting PCA on the items for perceived cause and consequences of the SME, reveals a subset of core variables which are different categories of psychological factors that may be associated with support for change. The items in each category are averaged to form composite variables measuring different categories of perceived causes and consequences, and associations between these items and different actions will be explored in the regression analyses that follows in the next section. Each of the perceived cause and consequence factors from the PCA therefore constitute an important part of the overall structure the mental models for transformative, policy and behaviour change.

- What are different mental models of transformative change, policy acceptability and willingness to change behaviours?

In this study, mental models are patterns of associations between causal beliefs that structure public support for transformative, policy and behaviour change. These patterns of associations are explored through regression analyses (detailed in the next section). The composite variables measuring categories of perceived causes and consequences obtained through the PCA act as predictor variables, along with others measured in the survey (e.g. perceived risk, controllability) and composite variables measuring policy and behaviour change obtained through the PCA act as outcome variables. Given the novel context of this study, i.e., to explore the mental models transformative change (including behaviours and policies) to address the SME (which has not been studied so far), we adopt standard PCA techniques because doing so has the advantage of enabling some comparisons to the previous literature, while being relatively straightforward to implement. For example, the PCA technique has been used for the similar purposes in related studies in environmental and sustainability psychology research to study mental models of solutions to climate change (for e.g. Bostrom et al.^3^). That said, in this study, the additional complexity in applying the PCA technique emerges from the wide range of items being used to measure the behavioural and policy support variables, as well as the causal and consequence belief variables, that emerged from the expert interviews and the scientific literature. For this reason, the PCA results from all four variable categories (i.e., cause and consequence beliefs, and behaviour and policy change) are presented in detail in this section. All data analyses were conducted in Stata 16 using the *factor, pcf* command, which specifies that the principal-component factor method be used to analyse the correlation matrix of the unweighted responses according to the Kaiser criterion (Eigenvalue > 1). Scree plots, factor loading plots and scoreplots are used to visually plot and analyse the data as well and are also presented in this section. The criterion for inclusion of an item in a component was first and foremost the factor loading. The two cases where the author’s judgement was used was when a) if the loadings were similar and b) if it made more theoretical sense to group an item in a particular component in relation to the other items in the cluster. The substitution of individual items for the factors in the regression equations changes significantly neither the statistical results nor our interpretation of the results. The author interpreted items in the cluster to give them a label, but there is often an overlap across components due to relatively similar or overlapping factor loadings. For example, where food and water availability can both be thought of ecological impacts, food availability was placed in lifestyle impacts as the factor loading was higher. Similarly, although active travel could also be a consumer behaviour, it was placed in waste behaviours due to the factor loading value.

**Perceived causes of the SME**

Table S 3: Principal Components Factor analysis with varimax rotation: Causal beliefs

| Factor | Variance | Difference | Proportion | Cumulative |
| --- | --- | --- | --- | --- |
| Factor1 | 4.66423 | 1.60019 | 0.3332 | 0.3332 |
| Factor2 | 3.06404 | . | 0.2189 | 0.552 |
| LR test: independent vs. saturated: chi2(91) = 4340.16 Prob>chi2 = 0.0000 | | | | |

Note: The scree test suggests two factors.

Figure S 5: Scree Plot

Note: The scree plot suggests two factors capture most of the variance in the data.

Table S 4: Rotated factor loadings (pattern matrix) and unique variances: Cause

| Variable | Factor1- Internal cause | Factor2-External cause | Uniqueness |
| --- | --- | --- | --- |
| Land-use change | 0.792 |  | 0.372 |
| Illegal extraction of animals and plants | 0.534 | 0.423 | 0.536 |
| Legal extraction of animals and plants | 0.730 |  | 0.441 |
| Climate change | 0.741 |  | 0.425 |
| Pests | 0.387 | 0.640 | 0.442 |
| Growth of the human population | 0.620 |  | 0.592 |
| Excess consumption and wasteful lifestyles | 0.737 |  | 0.392 |
| Pollution and toxins from producing goods and services | 0.730 |  | 0.402 |
| Economic incentives and policies leading to unsustainable practices | 0.784 |  | 0.347 |
| Political conflict | | 0.714 | 0.442 |
| Historical events | | 0.629 | 0.560 |
| Extreme weather events | | 0.779 | 0.392 |
| Animal viruses | | 0.767 | 0.383 |
| Co-extinctions | 0.569 | 0.358 | 0.548 |

Notes: Internal and External cause items corresponding to components 1 and 2 are in grey and white cells respectively.

Figure S 6: Loading plot

Note: Loading plot display which items account for the largest variation in each factor, and how they relate to each other. The graph shows that for Factor 1 (Internal cause) items such as land-use change and legal extraction of plants and animals account for most variance, whereas in Factor 2 (External cause) Extreme weather events and animal viruses account for most of the variance.

Figure S 7: Scoreplot

Note: Score plots approach the view of the loading matrix from the perspective of the observations.

## Perceived Consequences

Table S 5: Factor analysis/correlation with varimax rotation: Cause

| Factor | Variance | Difference | Proportion | Cumulative |
| --- | --- | --- | --- | --- |
| Factor1 | 7.19448 | 2.52066 | 0.2998 | 0.2998 |
| Factor2 | 4.67382 | 0.03824 | 0.1947 | 0.4945 |
| Factor3 | 4.63558 | . | 0.1931 | 0.6877 |
| LR test: independent vs. saturated: chi2(276) = 1.6e+04 Prob>chi2 = 0.0000 | | | | |

Note: The scree test suggests three factors.

Figure S 8: Screeplot

Note: The scree plot suggests three factors capture most of the variance in the data.

Table S 6: Rotated factor loadings (pattern matrix) and unique variances: Cause

| Variable | Factor1- Eco-impacts | Factor2-Lifestyle Impacts | Factor3-Disruptive Impacts | Uniqueness |
| --- | --- | --- | --- | --- |
| Food availability_UK | 0.508 | **0.6078** |  | 0.3604 |
| Water availability_UK | **0.6909** |  | 0.3723 | 0.3289 |
| Local culture and knowledge-UK | 0.6571 | **0.3809** | 0.422 |  |
| Mental health and well-being_UK | 0.3297 | **0.6058** | 0.4145 | 0.3525 |
| Economic growth & prosperity_UK | 0.4552 | 0.4581 | **0.4794** | 0.3531 |
| Displacement and migration_UK | 0.3349 |  | **0.6678** | 0.365 |
| Risk of diseases and pandemics_UK | **0.617** | 0.3863 |  | 0.3865 |
| Quality of life (e.g. living standards, consumption)_UK | 0.4678 | **0.5225** | 0.4534 | 0.3025 |
| Political conflict_UK | 0.3461 |  | **0.6924** | 0.3154 |
| Technological innovation_UK |  |  | **0.8084** | 0.2606 |
| Risk of extreme events (e.g. floods, hurricanes)_UK | **0.7596** |  | 0.3516 | 0.2843 |
| Climate change (e.g. global warming)_UK | **0.8066** |  |  | 0.275 |
| Food availability_Not UK | 0.6057 | **0.6231** |  | 0.2395 |
| Water availability_Not UK | **0.8012** |  |  | 0.205 |
| Local culture and knowledge-Not UK | 0.7236 |  | 0.374 |  |
| Mental health and well-being_Not UK | 0.349 | **0.6318** | 0.4175 | 0.3048 |
| Economic growth & prosperity_Not UK | 0.4937 | **0.5498** | 0.3684 | 0.3182 |
| Displacement and migration_Not UK | 0.4499 | 0.4114 | **0.4366** | 0.4377 |
| Risk of diseases and pandemics_Not UK | **0.6665** | 0.4123 |  | 0.3017 |
| Quality of life (e.g. living standards, consumption)_Not UK | 0.5931 | **0.523** | 0.3039 | 0.2824 |
| Political conflict_Not UK | 0.4516 | 0.3777 | **0.5811** | 0.3157 |
| Technological innovation_Not UK |  |  | **0.7787** | 0.269 |
| Risk of extreme events (e.g. floods, hurricanes)_Not UK | **0.8099** |  | 0.3171 | 0.2236 |
| Climate change (e.g. global warming)_Not UK | **0.8354** |  |  | 0.2184 |

Notes: Eco-, Lifestyle and Disruptive impact items corresponding to components 1, 2 and 3 are in grey, blue and white cells respectively.

Figure S 9: Loading plot

 Note: Loading plot display which items account for the largest variation in each factor, and how they relate to each other. The graph shows that for Factor 1 (eco impacts) extreme weather events and water availability account for a high share of variance, in Factor 2 (Lifestyle impacts)

Figure S 10: Scoreplots

Note: Score plots approach the view of the loading matrix from the perspective of the observations.

## Policy Acceptability

Table S 7: Factor analysis/correlation with varimax rotation: Policy Acceptability

| Factor | Variance | Difference | Proportion | Cumulative |
| --- | --- | --- | --- | --- |
| Factor1 | 7.36129 | 1.50654 | 0.2454 | 0.2454 |
| Factor2 | 5.85475 | 1.70002 | 0.1952 | 0.4405 |
| Factor3 | 4.15473 | 2.63863 | 0.1385 | 0.579 |
| Factor4 | 1.5161 | . | 0.0505 | 0.6296 |
| LR test: independent vs. saturated: chi2(435) = 1.5e+04 Prob>chi2 = 0.0000 | | | | |

Note: The scree test suggests four factors.

Figure S 11: Screeplot

Table S 8: Rotated factor loadings (pattern matrix) and unique variances: Policy Acceptability

| Variable | Factor1-EDM | Factor2-GLPP | Factor3-CEE | Factor4-TI | Uniqueness |
| --- | --- | --- | --- | --- | --- |
| Ban new fossil fuel licenses (e.g., oil, coal) | 0.3467 | **0.539** | 0.5063 |  | 0.3191 |
| Remove fossil fuel subsidies |  | **0.55** | 0.4351 |  | 0.3742 |
| Transition energy systems to renewables | 0.4328 | 0.3022 | **0.5709** |  | 0.3425 |
| Transition energy systems to nuclear energy |  |  |  | **0.8788** | 0.2129 |
| Re-wild natural and agricultural landscapes | **0.6845** |  | 0.3093 |  | 0.3675 |
| Stop industrial harvesting (e.g. trawling) in protected areas | 0.7009 | **0.3279** |  |  | 0.3752 |
| Mandate eco-certification for companies and eco-labels for consumers | 0.423 | **0.4968** | 0.4763 |  | 0.3262 |
| Prevent deforestation in recognised Indigenous territories | **0.7026** |  |  |  | 0.3738 |
| Enforce, manage and monitor wild species, natural habitats, and landscapes | **0.7523** |  |  |  | 0.2871 |
| Regulate commercial advertisement persuading people to buy more stuff | 0.3221 | **0.575** | 0.3099 |  | 0.4688 |
| Expand community urban green spaces (e.g., neighbourhood gardens, urban agriculture) | **0.7477** |  | 0.3115 |  | 0.3217 |
| Mandate carbon offsets for air travel |  | **0.7496** |  |  | 0.3063 |
| Carbon taxes for air travel |  | **0.7951** |  |  | 0.2906 |
| Four-day work week (longer hours/day+no pay reduction) |  |  | **0.6966** |  | 0.4412 |
| Frequent flyer and business class flight levies | 0.3076 | **0.7142** |  |  | 0.3824 |
| Green home (e.g. insulation, retrofitting) & electric vehicle subsidies | 0.4731 |  | **0.5219** |  | 0.3663 |
| Active travel subsidies (e.g. bike schemes) | 0.4841 |  | **0.5414** |  | 0.393 |
| Carbon tax meat |  | **0.6998** |  |  | 0.3847 |
| Invest in shared community-based resources (e.g. micro-grids) | **0.4861** | 0.4148 | 0.4119 |  | 0.4017 |
| Include sixth mass extinction in school and university curricula | **0.5253** | 0.4333 | 0.3795 |  | 0.3814 |
| Conduct public deliberation about extinction and biodiversity loss to gauge policy preferences | **0.5029** | 0.4303 | 0.3796 |  | 0.3962 |
| Wealth and carbon taxes on the super-rich |  | 0.4715 | **0.5305** |  | 0.4192 |
| Provide monetary compensation to indigenous and local communities to protect tropical forests | **0.5243** | 0.4661 |  |  | 0.4371 |
| Higher eco-taxes on polluting and extractive industries (e.g. multinational fossil fuel and agricultural companies) | 0.4343 | 0.4776 | **0.5028** |  | 0.3284 |
| Limit population growth humanely (e.g. encourage contraception use) | 0.3031 | **0.5583** |  |  | 0.5099 |
| Plant more trees in urban and degraded spaces | **0.7382** |  |  |  | 0.4038 |
| Geo-engineering | 0.3808 | 0.3479 | 0.4386 | **0.4203** | 0.3649 |
| Establish green buildings | **0.6551** |  | 0.3528 |  | 0.3823 |
| Establish animal and plant gene-banks to preserve genetic diversity | **0.6448** |  |  | 0.3274 | 0.4014 |
| Ban harmful products and pesticides | **0.6885** | 0.3717 |  |  | 0.3534 |

Notes: Conservation, market, lifestyle and big technology policy items corresponding to components 1, 2, 3 and 4 are in grey, blue, green and white cells respectively.

Figure S 12: Loading plot

Note: Loading plot display which items account for the largest variation in each factor, and how they relate to each other.

Figure S 13: scoreplot

Note: Score plots approach the view of the loading matrix from the perspective of the observations.

## Behaviour change

Table S 9: Factor analysis/correlation with varimax rotation: Policy Acceptability

| Factor | Variance | Difference | Proportion | Cumulative |
| --- | --- | --- | --- | --- |
| Factor1 | 7.361 | 1.507 | 0.245 | 0.245 |
| Factor2 | 5.855 | 1.700 | 0.195 | 0.441 |
| Factor3 | 4.155 | 2.639 | 0.139 | 0.579 |
| Factor4 | 1.516 | . | 0.051 | 0.630 |
| LR test: independent vs. saturated: chi2(435) = 1.5e+04 Prob>chi2 = 0.0000 | | | | |

Note: The scree test suggests four factors.

Figure S 14:Screeplot

Table S 10: Rotated factor loadings (pattern matrix) and unique variances: Policy Acceptability

|  | Factor1-Citizen | Factor2-Consumer | Factor3-Waste | Factor4-Nuclear | Uniqueness |
| --- | --- | --- | --- | --- | --- |
| Invest in energy and water efficiency devices | 0.4567 |  | **0.499** |  | 0.4493 |
| Use renewable energy in your home | 0.3548 |  | **0.6613** |  | 0.3607 |
| Reduce water use | 0.3114 |  | **0.6189** |  | 0.4079 |
| Nuclear |  |  |  | **0.831** | 0.3036 |
| Reduce intake of meat and animal proteins | 0.3066 | **0.7177** | 0.305 |  | 0.2953 |
| Waste less food |  |  | **0.7704** |  | 0.3174 |
| Recycle and sort waste |  |  | **0.7882** |  | 0.3268 |
| Adopt plant-based diets | 0.3431 | **0.7224** |  |  | 0.3173 |
| Work/study from home |  |  | **0.4331** |  | 0.7522 |
| Use active travel (e.g. walking and cycling) | 0.349 |  | **0.4841** |  | 0.58 |
| Avoid flights |  | **0.5785** |  |  | 0.5141 |
| Pay for carbon offsets and carbon taxes when flying | 0.4037 | **0.6075** |  |  | 0.3977 |
| Buy fewer things |  | 0.4808 | **0.5104** |  | 0.4178 |
| Have no or fewer children |  | **0.5558** |  |  | 0.5775 |
| Buy fewer animal products | 0.3186 | **0.7397** |  |  | 0.2699 |
| Buy sustainably sourced and eco-labelled products | 0.4624 | **0.4925** | 0.4203 |  | 0.3664 |
| Elect politicians committed to halting extinction and climate change | **0.5045** | 0.4617 | 0.3549 |  | 0.406 |
| Share resources with others | **0.5859** |  | 0.347 |  | 0.4476 |
| Support eco-protests and green petitions | **0.6817** | 0.4473 |  |  | 0.3076 |
| Participate in eco-movements | **0.7792** | 0.4174 |  |  | 0.2155 |
| Undergo professional retraining to enact sustainability at home and work | **0.7054** |  |  |  | 0.4425 |
| Spend time learning about extinction debates and issues | **0.7773** |  |  |  | 0.2837 |
| Talk to friends, family and colleagues about extinction | **0.7678** |  |  |  | 0.2654 |
| Provide habitats for wildlife in gardens/balconies | **0.4706** |  | 0.4587 |  | 0.5361 |
| Volunteer time to restore nature | **0.7495** |  |  |  | 0.3521 |
| Donate money to conservation projects & supporting wild animals | **0.7325** |  |  |  | 0.3802 |
| Participate in ecological citizen science projects | **0.8133** |  |  |  | 0.2477 |

Notes: Citizenship, consumer, waste and nuclear behavioural items corresponding to components 1, 2, 3 and 4 are in grey, blue, green and white cells respectively.

Figure S 15: Loading plot

Note: Loading plot display which items account for the largest variation in each factor, and how they relate to each other.

Figure S 16: Scoreplot

Note: Score plots approach the view of the loading matrix from the perspective of the observations.

Table S 11: Summary of the composite variables of perceived causes, consequences, policy acceptance and willingness-to-change behaviours based on component factors derived from the PCA

| **Category** | **Factor groups (mean, SD)** | **Items** | **Cronbach’s alpha** |
| --- | --- | --- | --- |
| Causes | Internal  5.560 (0.948) | Land-use change; Illegal extraction of animals and plants; Legal industrial extraction of animals and plants; Climate change; Co-extinctions; Economic incentives and policies leading to unsustainable practices; Pollution and toxins from producing goods and services; Excess consumption and wasteful lifestyles; Growth of the human population | 0.889 |
|  | External  4.234 (1.139) | Harmful pests and viruses bought in by humans; Wars and armed conflicts; Historical events; Extreme events; Viruses passed between animals | 0.792 |
| Consequences | Eco-Impact  5.167 (1.411) | Risk of extreme events-UK; Climate change-UK; Risk of diseases and pandemics-UK, Risk of extreme events-Non-UK; Climate Change-Non-UK; Risk of diseases and pandemics-Non-UK, Water availability-UK, Water Availability-Non-UK | 0.944 |
|  | Lifestyle Impact  5.083 (1.196) | Food availability-UK; Local culture and knowledge-UK; Mental health and well-being-UK; Economic growth & prosperity-UK, Food Availability-Non-UK; Local culture and knowledge-Non-UK; Mental health and well-being-Non-UK; Economic growth & prosperity-Non-UK, Quality of life-UK, Quality of life-Non-UK | 0.931 |
|  | Disruptive Impact  4.388 (1.425) | Technological innovation-UK; Political conflict-UK; Displacement and migration of residents-UK, Technological innovation-non-UK; Political conflict-non-UK; Displacement and migration of residents-non-UK | 0.901 |
| Policies | Conservation  5.881 (1.033) | Plant more trees in urban and degraded spaces; Ban harmful products and pesticides; Establish animal and plant gene-banks to preserve genetic diversity; Establish green buildings; Provide monetary compensation to indigenous and local communities to protect tropical forests; Conduct public deliberation about extinction and biodiversity loss to gauge policy preferences; Include sixth mass extinction in school and university curricula; Invest in shared community- based resources; Expand community urban green spaces; Enforce, manage and monitor wild species, natural habitats, and landscapes; Prevent deforestation in recognised Indigenous territories; Re-wild natural and agricultural landscapes | 0.9365 |
|  | Market  5.129 (1.370) | Ban new fossil fuel licences; Remove fossil fuel subsidies; Mandate eco-certification for companies and eco-labels for consumers; Regulate commercial advertisement persuading people to buy more stuff; Mandate carbon offsets for air travel; Impose carbon taxes for air travel; Impose frequent flyer and business class flight levies; Impose a carbon tax on meat; Humanely limit population growth, Stop industrial harvesting | 0.896 |
|  | Lifestyle  5.817 (1.164) | Transition energy systems to renewables; Institute a four-day work week; Provide green home & electric vehicle subsidies; Provide active travel subsidies; Impose wealth and carbon taxes on the superrich; Impose higher eco-taxes on polluting and extractive industries | 0.868 |
|  | Technology  5.189 (1.311) | Transition energy systems to nuclear energy; Adopt geo-engineering technologies | - |
| Behaviours | Citizenship  4.941 (1.383) | Talk to friends, family and colleagues about extinction; Volunteer time to restore nature; Donate money to conservation projects & supporting wild animals; Participate in ecological citizen science projects; Spend time learning about extinction debates and issues; Undergo professional retraining to enact sustainability at home and work; Participate in eco-movements; Support eco-protests and green petitions; Share resources with others; Elect politicians committed to halting extinction and climate change | 0.935 |
|  | Consumer  4.818 (1.432) | Reduce intake of meat and animal proteins; Adopt plant-based diets; Avoid flights; Pay for carbon offsets and carbon taxes when flying; Have no or fewer children; Buy fewer animal products; Buy sustainably sourced and eco-labelled products | 0.865 |
|  | Waste  5.933 (0.927) | Invest in energy and water efficiency devices; Use renewable energy in your home; Reduce water use; Waste less food; Recycle and sort waste; Work/study from home; Use active travel; Buy fewer things | 0.8522 |
|  | Nuclear energy  4.548 (1.843) | Transition to nuclear energy | - |

Note: The Cronbach’s alpha measure of scale reliability was generally above the conventional 0.6 standard for all multi-item components, indicating items in each component were closely related. N=739, min value = 1 and max value = 7. SD = Standard deviation.

# Supplementary note 5: Regression Analyses

Regression analyses were conducted to examine associations between different types of causal beliefs and support for transformative, policy and behaviour change. These patterns of associations are referred to as mental models of transformative, policy and behaviour change. Specifically, in this study, regression analyses were used to address the following questions:

- Is there diversity in mental models among the participants? Are there potential trade-offs between these mental models?

Here we identify associations between the outcome variables of support for transformative change, and different categories of policy and behaviour change identified from the PCA, with predictor variables measuring causal beliefs including different types of perceived causes and consequences (also derived from PCA), perceived effectiveness, perceived risk, perceived controllability, trust in science, and perceived disagreement amongst scientists. Whether the direction of associations between these psychological factors and support for change outcomes vary systematically is explored. Differences in the direction of associations between variables it is suggestive of trade-offs in some mental models of action. For e.g. if perceived effectiveness of citizenship behaviours is negatively associated with support for consumer behaviours but is positively associated with citizen behaviour change support.

- What participant characteristics are associated with support for change, in each of these mental models?

Here we identify associations between the outcome variables of support for transformative change, and policy and behaviour change, with self-reported characteristics of the participants (e.g., egoistic, socio-altruistic and biospheric values, personal experience of nature loss and socio-demographic data).

For the regression analyses, ordinary least squares regression with standardised variables and robust standard errors was used to obtain associations. We used the *reg* command with the *vce(r)* option. The number of observations drops to 602 (from 739) because 137 respondents skipped questions on perceptions of the science. Model 1 explores associations with support for change and causal beliefs and attitudes, whereas Model 2 aims to replicate the results from Model 1 while additionally controls for covariates measuring values, personal experiences and socio-demographic characteristics. The tables provide standardised coefficients. Standardizing variables allows for easier comparison of their effects in a regression model. Standardised coefficients indicate the change in the outcome variable (in standard deviation units) for a one standard deviation change in the predictor variable. It is useful when combining variables with different scales or units, as is the case in this study. The variance inflation factor was less than the conventional standard of 10 for all the estimated models, suggesting that there was no multicollinearity. All analyses were conducted in Stata 16. Results are detailed below for each of the outcomes, to supplement the coefficient plots presented in the main text.

Table S 13: Mental models of support for transformative change

| VARIABLES | Statistic | Model 1 | Model 2 |
| --- | --- | --- | --- |
| Internal cause | B | 0.530*** | 0.439*** |
|  | SE | (0.048) | (0.047) |
|  | p | 0.000 | 0.000 |
|  | 95% CI | (0.435 - 0.625) | (0.347 - 0.531) |
| External cause | B | -0.237*** | -0.201*** |
|  | SE | (0.039) | (0.038) |
|  | p | 0.000 | 0.000 |
|  | 95% CI | (-0.313 - -0.160) | (-0.275 - -0.127) |
| Eco impact | B | 0.044 | 0.029 |
|  | SE | (0.059) | (0.054) |
|  | p | 0.457 | 0.596 |
|  | 95% CI | (-0.071 - 0.159) | (-0.078 - 0.136) |
| Lifestyle impact | B | 0.274*** | 0.217*** |
|  | SE | (0.058) | (0.054) |
|  | p | 0.000 | 0.000 |
|  | 95% CI | (0.160 - 0.388) | (0.111 - 0.324) |
| Disrupt impact | B | -0.162*** | -0.156*** |
|  | SE | (0.060) | (0.056) |
|  | p | 0.007 | 0.006 |
|  | 95% CI | (-0.281 - -0.044) | (-0.266 - -0.045) |
| Controllable | B | 0.063* | 0.068** |
|  | SE | (0.038) | (0.034) |
|  | p | 0.098 | 0.042 |
|  | 95% CI | (-0.012 - 0.138) | (0.003 - 0.134) |
| Perceived risk | B | 0.139*** | 0.083** |
|  | SE | (0.038) | (0.039) |
|  | p | 0.000 | 0.034 |
|  | 95% CI | (0.064 - 0.214) | (0.006 - 0.159) |
| Trust scientists | B | 0.047 | 0.047 |
|  | SE | (0.033) | (0.031) |
|  | p | 0.149 | 0.127 |
|  | 95% CI | (-0.017 - 0.111) | (-0.013 - 0.107) |
| Scientists disagree | B | -0.106*** | -0.062* |
|  | SE | (0.034) | (0.032) |
|  | p | 0.002 | 0.052 |
|  | 95% CI | (-0.173 - -0.039) | (-0.124 - 0.001) |
| Past knowledge | B |  | 0.130*** |
|  | SE |  | (0.032) |
|  | p |  | 0.000 |
|  | 95% CI |  | (0.067 - 0.193) |
| Experience nature loss | B |  | 0.084*** |
|  | SE |  | (0.032) |
|  | p |  | 0.009 |
|  | 95% CI |  | (0.021 - 0.148) |
| Biospheric values | B |  | 0.043 |
|  | SE |  | (0.044) |
|  | p |  | 0.335 |
|  | 95% CI |  | (-0.044 - 0.129) |
| Socio-altruistic values | B |  | 0.174*** |
|  | SE |  | (0.041) |
|  | p |  | 0.000 |
|  | 95% CI |  | (0.094 - 0.255) |
| Egoistic values | B |  | -0.166*** |
|  | SE |  | (0.030) |
|  | p |  | 0.000 |
|  | 95% CI |  | (-0.225 - -0.106) |
| Female+other | B |  | -0.080 |
|  | SE |  | (0.057) |
|  | p |  | 0.158 |
|  | 95% CI |  | (-0.191 - 0.031) |
| Not white | B |  | 0.110 |
|  | SE |  | (0.080) |
|  | p |  | 0.169 |
|  | 95% CI |  | (-0.047 - 0.267) |
| Age | B |  | 0.048* |
|  | SE |  | (0.028) |
|  | p |  | 0.094 |
|  | 95% CI |  | (-0.008 - 0.104) |
| Income | B |  | -0.031 |
|  | SE |  | (0.029) |
|  | p |  | 0.288 |
|  | 95% CI |  | (-0.088 - 0.026) |
| Education | B |  | 0.049* |
|  | SE |  | (0.028) |
|  | p |  | 0.081 |
|  | 95% CI |  | (-0.006 - 0.105) |
| Constant | B | 5.317*** | 5.336*** |
|  | SE | (0.030) | (0.040) |
|  | p | 0.000 | 0.000 |
|  | 95% CI | (5.259 - 5.375) | (5.257 - 5.415) |
| Observations |  | 602 | 602 |
| R-squared |  | 0.508 | 0.593 |
| Covariates |  | No | No |

Results from Ordinary Least Squares regression analyses. Robust SE (standard error) and CI (confidence interval in parentheses, B is the standardised coefficient, *** p<0.01, ** p<0.05, * p<0.1.

Table S 14: Conservation policies mental model:

| VARIABLES | Statistic | Model 1 | Model 2 |
| --- | --- | --- | --- |
| Internal cause | B | 0.534*** | 0.442*** |
|  | SE | (0.041) | (0.044) |
|  | p | 0.000 | 0.000 |
|  | 95% CI | (0.453 - 0.615) | (0.355 - 0.528) |
| External cause | B | -0.157*** | -0.117*** |
|  | SE | (0.029) | (0.030) |
|  | p | 0.000 | 0.000 |
|  | 95% CI | (-0.214 - -0.100) | (-0.176 - -0.057) |
| Eco impact | B | 0.004 | -0.006 |
|  | SE | (0.045) | (0.043) |
|  | p | 0.925 | 0.887 |
|  | 95% CI | (-0.084 - 0.093) | (-0.090 - 0.078) |
| Lifestyle impact | B | 0.182*** | 0.141** |
|  | SE | (0.058) | (0.057) |
|  | p | 0.002 | 0.013 |
|  | 95% CI | (0.068 - 0.295) | (0.030 - 0.253) |
| Disrupt impact | B | -0.070 | -0.068 |
|  | SE | (0.051) | (0.046) |
|  | p | 0.167 | 0.144 |
|  | 95% CI | (-0.170 - 0.029) | (-0.158 - 0.023) |
| Conservation policy effective | B | 0.430*** | 0.362*** |
|  | SE | (0.061) | (0.062) |
|  | p | 0.000 | 0.000 |
|  | 95% CI | (0.310 - 0.549) | (0.241 - 0.484) |
| Market policy effective | B | 0.060 | 0.014 |
|  | SE | (0.054) | (0.055) |
|  | p | 0.273 | 0.799 |
|  | 95% CI | (-0.047 - 0.167) | (-0.093 - 0.121) |
| Lifestyle policy effective | B | -0.174*** | -0.125** |
|  | SE | (0.065) | (0.062) |
|  | p | 0.007 | 0.046 |
|  | 95% CI | (-0.302 - -0.047) | (-0.247 - -0.002) |
| Big-tech policy effective | B | -0.075* | -0.032 |
|  | SE | (0.040) | (0.040) |
|  | p | 0.060 | 0.434 |
|  | 95% CI | (-0.153 - 0.003) | (-0.111 - 0.048) |
| Controllable | B | 0.011 | 0.009 |
|  | SE | (0.031) | (0.027) |
|  | p | 0.715 | 0.740 |
|  | 95% CI | (-0.049 - 0.072) | (-0.045 - 0.063) |
| Perceived risk | B | 0.120*** | 0.066** |
|  | SE | (0.035) | (0.033) |
|  | p | 0.001 | 0.046 |
|  | 95% CI | (0.052 - 0.188) | (0.001 - 0.132) |
| Trust scientists | B | 0.042 | 0.048* |
|  | SE | (0.029) | (0.029) |
|  | p | 0.151 | 0.098 |
|  | 95% CI | (-0.015 - 0.100) | (-0.009 - 0.104) |
| Scientists disagree | B | -0.026 | -0.022 |
|  | SE | (0.027) | (0.025) |
|  | p | 0.329 | 0.385 |
|  | 95% CI | (-0.079 - 0.027) | (-0.070 - 0.027) |
| Past knowledge | B |  | 0.040 |
|  | SE |  | (0.027) |
|  | p |  | 0.145 |
|  | 95% CI |  | (-0.014 - 0.093) |
| Experience nature loss | B |  | -0.004 |
|  | SE |  | (0.027) |
|  | p |  | 0.874 |
|  | 95% CI |  | (-0.056 - 0.048) |
| Experience extreme events | B |  | -0.022 |
|  | SE |  | (0.025) |
|  | p |  | 0.385 |
|  | 95% CI |  | (-0.072 - 0.028) |
| Biospheric values | B |  | 0.230*** |
|  | SE |  | (0.040) |
|  | p |  | 0.000 |
|  | 95% CI |  | (0.152 - 0.307) |
| Socio-altruistic values | B |  | 0.041 |
|  | SE |  | (0.038) |
|  | p |  | 0.276 |
|  | 95% CI |  | (-0.033 - 0.115) |
| Egoistic values | B |  | -0.051* |
|  | SE |  | (0.027) |
|  | p |  | 0.064 |
|  | 95% CI |  | (-0.104 - 0.003) |
| Female+other | B |  | 0.026 |
|  | SE |  | (0.051) |
|  | p |  | 0.617 |
|  | 95% CI |  | (-0.075 - 0.126) |
| Not white | B |  | -0.072 |
|  | SE |  | (0.075) |
|  | p |  | 0.338 |
|  | 95% CI |  | (-0.218 - 0.075) |
| Age | B |  | -0.000 |
|  | SE |  | (0.026) |
|  | p |  | 0.994 |
|  | 95% CI |  | (-0.052 - 0.051) |
| Income | B |  | -0.004 |
|  | SE |  | (0.024) |
|  | p |  | 0.874 |
|  | 95% CI |  | (-0.051 - 0.043) |
| Education | B |  | 0.033 |
|  | SE |  | (0.024) |
|  | p |  | 0.172 |
|  | 95% CI |  | (-0.014 - 0.081) |
| Constant | B | -0.012 | -0.017 |
|  | SE | (0.025) | (0.037) |
|  | p | 0.617 | 0.647 |
|  | 95% CI | (-0.060 - 0.036) | (-0.090 - 0.056) |
|  |  |  |  |
| Observations |  | 602 | 602 |
| R-squared |  | 0.643 | 0.691 |
| Covariates |  | No | Yes |

Results from Ordinary Least Squares regression analyses. Robust SE (standard error) and CI (confidence interval in parentheses, B is the standardised coefficient, *** p<0.01, ** p<0.05, * p<0.1.

Table S 15:Market policies mental model:

| VARIABLES | Statistic | Model 1 | Model 2 |
| --- | --- | --- | --- |
| Internal cause | B | 0.358*** | 0.296*** |
|  | SE | (0.034) | (0.037) |
|  | p | 0.000 | 0.000 |
|  | 95% CI | (0.290 - 0.425) | (0.224 - 0.368) |
| External cause | B | -0.052* | -0.029 |
|  | SE | (0.028) | (0.029) |
|  | p | 0.062 | 0.310 |
|  | 95% CI | (-0.108 - 0.003) | (-0.086 - 0.027) |
| Eco impact | B | 0.036 | 0.011 |
|  | SE | (0.043) | (0.042) |
|  | p | 0.401 | 0.789 |
|  | 95% CI | (-0.048 - 0.120) | (-0.072 - 0.095) |
| Lifestyle impact | B | 0.162*** | 0.141*** |
|  | SE | (0.051) | (0.051) |
|  | p | 0.001 | 0.006 |
|  | 95% CI | (0.062 - 0.262) | (0.041 - 0.241) |
| Disrupt impact | B | -0.042 | -0.038 |
|  | SE | (0.042) | (0.041) |
|  | p | 0.325 | 0.355 |
|  | 95% CI | (-0.125 - 0.041) | (-0.118 - 0.043) |
| Conservation policy effective | B | -0.255*** | -0.303*** |
|  | SE | (0.059) | (0.060) |
|  | p | 0.000 | 0.000 |
|  | 95% CI | (-0.371 - -0.138) | (-0.420 - -0.186) |
| Market policy effective | B | 0.831*** | 0.795*** |
|  | SE | (0.056) | (0.056) |
|  | p | 0.000 | 0.000 |
|  | 95% CI | (0.721 - 0.942) | (0.685 - 0.905) |
| Lifestyle policy effective | B | -0.130** | -0.094 |
|  | SE | (0.064) | (0.062) |
|  | p | 0.042 | 0.126 |
|  | 95% CI | (-0.255 - -0.005) | (-0.215 - 0.026) |
| Big-tech policy effective | B | -0.108*** | -0.071* |
|  | SE | (0.037) | (0.039) |
|  | p | 0.004 | 0.067 |
|  | 95% CI | (-0.182 - -0.035) | (-0.147 - 0.005) |
| Controllable | B | 0.011 | 0.011 |
|  | SE | (0.028) | (0.026) |
|  | p | 0.685 | 0.661 |
|  | 95% CI | (-0.043 - 0.065) | (-0.040 - 0.062) |
| Perceived risk | B | 0.084*** | 0.051* |
|  | SE | (0.031) | (0.030) |
|  | p | 0.006 | 0.092 |
|  | 95% CI | (0.024 - 0.144) | (-0.008 - 0.110) |
| Trust scientists | B | 0.071*** | 0.079*** |
|  | SE | (0.026) | (0.025) |
|  | p | 0.006 | 0.002 |
|  | 95% CI | (0.020 - 0.122) | (0.029 - 0.129) |
| Scientists disagree | B | -0.018 | -0.010 |
|  | SE | (0.026) | (0.025) |
|  | p | 0.484 | 0.685 |
|  | 95% CI | (-0.068 - 0.032) | (-0.058 - 0.038) |
| Past knowledge | B |  | 0.014 |
|  | SE |  | (0.023) |
|  | p |  | 0.540 |
|  | 95% CI |  | (-0.031 - 0.060) |
| Experience nature loss | B |  | 0.016 |
|  | SE |  | (0.025) |
|  | p |  | 0.539 |
|  | 95% CI |  | (-0.034 - 0.065) |
| Experience extreme events | B |  | 0.001 |
|  | SE |  | (0.026) |
|  | p |  | 0.955 |
|  | 95% CI |  | (-0.049 - 0.052) |
| Biospheric values | B |  | 0.144*** |
|  | SE |  | (0.036) |
|  | p |  | 0.000 |
|  | 95% CI |  | (0.074 - 0.214) |
| Socio-altruistic values | B |  | 0.039 |
|  | SE |  | (0.036) |
|  | p |  | 0.282 |
|  | 95% CI |  | (-0.032 - 0.109) |
| Egoistic values | B |  | -0.053** |
|  | SE |  | (0.025) |
|  | p |  | 0.032 |
|  | 95% CI |  | (-0.102 - -0.005) |
| Female+other | B |  | 0.006 |
|  | SE |  | (0.048) |
|  | p |  | 0.902 |
|  | 95% CI |  | (-0.088 - 0.100) |
| Not white | B |  | 0.009 |
|  | SE |  | (0.065) |
|  | p |  | 0.890 |
|  | 95% CI |  | (-0.119 - 0.137) |
| Age | B |  | 0.030 |
|  | SE |  | (0.023) |
|  | p |  | 0.184 |
|  | 95% CI |  | (-0.014 - 0.075) |
| Income | B |  | -0.009 |
|  | SE |  | (0.022) |
|  | p |  | 0.678 |
|  | 95% CI |  | (-0.052 - 0.034) |
| Education | B |  | 0.042* |
|  | SE |  | (0.024) |
|  | p |  | 0.076 |
|  | 95% CI |  | (-0.004 - 0.089) |
| Constant | B | 0.002 | -0.003 |
|  | SE | (0.022) | (0.035) |
|  | p | 0.931 | 0.939 |
|  | 95% CI | (-0.042 - 0.045) | (-0.072 - 0.066) |
|  |  |  |  |
| Observations |  | 602 | 602 |
| R-squared |  | 0.711 | 0.736 |
| Covariates |  | No | Yes |

Results from Ordinary Least Squares regression analyses. Robust SE (standard error) and CI (confidence interval in parentheses, B is the standardised coefficient, *** p<0.01, ** p<0.05, * p<0.1.

Table S 16: Lifestyle policies mental model:

| VARIABLES | Statistic | Model 1 | Model 2 |
| --- | --- | --- | --- |
| Internal cause | B | 0.422*** | 0.381*** |
|  | SE | (0.047) | (0.049) |
|  | p | 0.000 | 0.000 |
|  | 95% CI | (0.329 - 0.515) | (0.284 - 0.478) |
| External cause | B | -0.108*** | -0.097*** |
|  | SE | (0.032) | (0.034) |
|  | p | 0.001 | 0.005 |
|  | 95% CI | (-0.171 - -0.045) | (-0.164 - -0.030) |
| Eco impact | B | 0.033 | 0.028 |
|  | SE | (0.050) | (0.052) |
|  | p | 0.515 | 0.586 |
|  | 95% CI | (-0.066 - 0.131) | (-0.073 - 0.129) |
| Lifestyle impact | B | 0.157** | 0.120* |
|  | SE | (0.067) | (0.067) |
|  | p | 0.020 | 0.074 |
|  | 95% CI | (0.024 - 0.290) | (-0.012 - 0.252) |
| Disrupt impact | B | -0.077 | -0.070 |
|  | SE | (0.056) | (0.056) |
|  | p | 0.170 | 0.213 |
|  | 95% CI | (-0.188 - 0.033) | (-0.181 - 0.040) |
| Conservation policy effective | B | -0.231*** | -0.269*** |
|  | SE | (0.089) | (0.090) |
|  | p | 0.010 | 0.003 |
|  | 95% CI | (-0.407 - -0.056) | (-0.447 - -0.092) |
| Market policy effective | B | 0.087 | 0.069 |
|  | SE | (0.065) | (0.068) |
|  | p | 0.181 | 0.310 |
|  | 95% CI | (-0.041 - 0.215) | (-0.064 - 0.201) |
| Lifestyle policy effective | B | 0.508*** | 0.517*** |
|  | SE | (0.094) | (0.090) |
|  | p | 0.000 | 0.000 |
|  | 95% CI | (0.324 - 0.692) | (0.339 - 0.694) |
| Big-tech policy effective | B | -0.088* | -0.064 |
|  | SE | (0.045) | (0.048) |
|  | p | 0.054 | 0.185 |
|  | 95% CI | (-0.177 - 0.002) | (-0.159 - 0.031) |
| Controllable | B | 0.036 | 0.029 |
|  | SE | (0.036) | (0.036) |
|  | p | 0.320 | 0.413 |
|  | 95% CI | (-0.035 - 0.107) | (-0.041 - 0.099) |
| Perceived risk | B | 0.108*** | 0.074* |
|  | SE | (0.039) | (0.041) |
|  | p | 0.006 | 0.068 |
|  | 95% CI | (0.031 - 0.184) | (-0.006 - 0.154) |
| Trust scientists | B | 0.055 | 0.047 |
|  | SE | (0.036) | (0.036) |
|  | p | 0.123 | 0.203 |
|  | 95% CI | (-0.015 - 0.126) | (-0.025 - 0.118) |
| Scientists disagree | B | -0.054* | -0.054* |
|  | SE | (0.032) | (0.032) |
|  | p | 0.088 | 0.098 |
|  | 95% CI | (-0.117 - 0.008) | (-0.117 - 0.010) |
| Past knowledge | B |  | 0.007 |
|  | SE |  | (0.030) |
|  | p |  | 0.824 |
|  | 95% CI |  | (-0.053 - 0.066) |
| Experience nature loss | B |  | -0.028 |
|  | SE |  | (0.034) |
|  | p |  | 0.421 |
|  | 95% CI |  | (-0.095 - 0.040) |
| Experience extreme events | B |  | -0.003 |
|  | SE |  | (0.029) |
|  | p |  | 0.931 |
|  | 95% CI |  | (-0.060 - 0.055) |
| Biospheric values | B |  | 0.102** |
|  | SE |  | (0.046) |
|  | p |  | 0.027 |
|  | 95% CI |  | (0.012 - 0.193) |
| Socio-altruistic values | B |  | 0.113** |
|  | SE |  | (0.045) |
|  | p |  | 0.012 |
|  | 95% CI |  | (0.025 - 0.202) |
| Egoistic values | B |  | -0.025 |
|  | SE |  | (0.029) |
|  | p |  | 0.401 |
|  | 95% CI |  | (-0.083 - 0.033) |
| Female+other | B |  | 0.002 |
|  | SE |  | (0.056) |
|  | p |  | 0.978 |
|  | 95% CI |  | (-0.108 - 0.111) |
| Not white | B |  | -0.070 |
|  | SE |  | (0.080) |
|  | p |  | 0.385 |
|  | 95% CI |  | (-0.227 - 0.088) |
| Age | B |  | -0.015 |
|  | SE |  | (0.029) |
|  | p |  | 0.611 |
|  | 95% CI |  | (-0.071 - 0.042) |
| Income | B |  | 0.011 |
|  | SE |  | (0.029) |
|  | p |  | 0.713 |
|  | 95% CI |  | (-0.047 - 0.068) |
| Education | B |  | 0.024 |
|  | SE |  | (0.028) |
|  | p |  | 0.385 |
|  | 95% CI |  | (-0.030 - 0.078) |
| Constant | B | -0.014 | -0.004 |
|  | SE | (0.027) | (0.043) |
|  | p | 0.602 | 0.932 |
|  | 95% CI | (-0.067 - 0.039) | (-0.089 - 0.082) |
|  |  |  |  |
| Observations |  | 602 | 602 |
| R-squared |  | 0.597 | 0.618 |
| Covariates |  | No | Yes |

Results from Ordinary Least Squares regression analyses. Robust SE (standard error) and CI (confidence interval in parentheses, B is the standardised coefficient, *** p<0.01, ** p<0.05, * p<0.1.

Table S 17: Big-tech policies mental model:

| VARIABLES | Statistic | Model 1 | Model 2 |
| --- | --- | --- | --- |
| Internal cause | B | 0.221*** | 0.200*** |
|  | SE | (0.055) | (0.056) |
|  | p | 0.000 | 0.000 |
|  | 95% CI | (0.112 - 0.329) | (0.090 - 0.310) |
| External cause | B | -0.030 | -0.007 |
|  | SE | (0.037) | (0.040) |
|  | p | 0.412 | 0.858 |
|  | 95% CI | (-0.103 - 0.042) | (-0.085 - 0.071) |
| Eco impact | B | -0.059 | -0.037 |
|  | SE | (0.057) | (0.060) |
|  | p | 0.298 | 0.541 |
|  | 95% CI | (-0.171 - 0.052) | (-0.154 - 0.081) |
| Lifestyle impact | B | 0.142** | 0.135* |
|  | SE | (0.071) | (0.072) |
|  | p | 0.047 | 0.059 |
|  | 95% CI | (0.002 - 0.282) | (-0.005 - 0.276) |
| Disrupt impact | B | -0.064 | -0.105* |
|  | SE | (0.058) | (0.059) |
|  | p | 0.268 | 0.073 |
|  | 95% CI | (-0.177 - 0.049) | (-0.220 - 0.010) |
| Conservation policy effective | B | -0.185** | -0.200** |
|  | SE | (0.075) | (0.080) |
|  | p | 0.014 | 0.013 |
|  | 95% CI | (-0.333 - -0.038) | (-0.357 - -0.043) |
| Market policy effective | B | 0.082 | 0.066 |
|  | SE | (0.075) | (0.073) |
|  | p | 0.279 | 0.368 |
|  | 95% CI | (-0.066 - 0.229) | (-0.078 - 0.209) |
| Lifestyle policy effective | B | -0.139 | -0.114 |
|  | SE | (0.085) | (0.086) |
|  | p | 0.103 | 0.184 |
|  | 95% CI | (-0.306 - 0.028) | (-0.283 - 0.054) |
| Big-tech policy effective | B | 0.735*** | 0.723*** |
|  | SE | (0.049) | (0.052) |
|  | p | 0.000 | 0.000 |
|  | 95% CI | (0.638 - 0.831) | (0.622 - 0.825) |
| Controllable | B | 0.032 | 0.021 |
|  | SE | (0.039) | (0.037) |
|  | p | 0.405 | 0.568 |
|  | 95% CI | (-0.044 - 0.108) | (-0.052 - 0.095) |
| Perceived risk | B | 0.054 | 0.043 |
|  | SE | (0.041) | (0.041) |
|  | p | 0.192 | 0.292 |
|  | 95% CI | (-0.027 - 0.134) | (-0.037 - 0.124) |
| Trust scientists | B | 0.051* | 0.041 |
|  | SE | (0.030) | (0.031) |
|  | p | 0.092 | 0.190 |
|  | 95% CI | (-0.008 - 0.110) | (-0.021 - 0.103) |
| Scientists disagree | B | -0.025 | -0.035 |
|  | SE | (0.032) | (0.032) |
|  | p | 0.431 | 0.281 |
|  | 95% CI | (-0.087 - 0.037) | (-0.098 - 0.029) |
| Past knowledge | B |  | 0.039 |
|  | SE |  | (0.032) |
|  | p |  | 0.215 |
|  | 95% CI |  | (-0.023 - 0.101) |
| Experience nature loss | B |  | -0.013 |
|  | SE |  | (0.034) |
|  | p |  | 0.709 |
|  | 95% CI |  | (-0.080 - 0.055) |
| Experience extreme events | B |  | -0.023 |
|  | SE |  | (0.030) |
|  | p |  | 0.434 |
|  | 95% CI |  | (-0.082 - 0.035) |
| Biospheric values | B |  | 0.080* |
|  | SE |  | (0.041) |
|  | p |  | 0.050 |
|  | 95% CI |  | (-0.000 - 0.159) |
| Socio-altruistic values | B |  | 0.000 |
|  | SE |  | (0.042) |
|  | p |  | 0.997 |
|  | 95% CI |  | (-0.083 - 0.083) |
| Egoistic values | B |  | 0.067** |
|  | SE |  | (0.032) |
|  | p |  | 0.033 |
|  | 95% CI |  | (0.005 - 0.130) |
| Female+other | B |  | -0.117* |
|  | SE |  | (0.065) |
|  | p |  | 0.071 |
|  | 95% CI |  | (-0.244 - 0.010) |
| Not white | B |  | -0.028 |
|  | SE |  | (0.089) |
|  | p |  | 0.750 |
|  | 95% CI |  | (-0.204 - 0.147) |
| Age | B |  | 0.009 |
|  | SE |  | (0.030) |
|  | p |  | 0.765 |
|  | 95% CI |  | (-0.051 - 0.069) |
| Income | B |  | 0.028 |
|  | SE |  | (0.030) |
|  | p |  | 0.351 |
|  | 95% CI |  | (-0.031 - 0.087) |
| Education | B |  | 0.027 |
|  | SE |  | (0.029) |
|  | p |  | 0.362 |
|  | 95% CI |  | (-0.031 - 0.084) |
| Constant | B | -0.005 | 0.056 |
|  | SE | (0.028) | (0.045) |
|  | p | 0.866 | 0.220 |
|  | 95% CI | (-0.060 - 0.050) | (-0.033 - 0.145) |
|  |  |  |  |
| Observations |  | 602 | 602 |
| R-squared |  | 0.544 | 0.560 |
| Covariates |  | No | Yes |

Results from Ordinary Least Squares regression analyses. Robust SE (standard error) and CI (confidence interval in parentheses, B is the standardised coefficient, *** p<0.01, ** p<0.05, * p<0.1.

Table S 18: Citizenship behaviours mental model:

| VARIABLES | Statistic | Model 1 | Model 1 |
| --- | --- | --- | --- |
| Internal cause | B | 0.135*** | 0.065 |
|  | SE | (0.039) | (0.040) |
|  | p | 0.001 | 0.104 |
|  | 95% CI | (0.058 - 0.212) | (-0.013 - 0.144) |
| External cause | B | -0.049 | -0.016 |
|  | SE | (0.035) | (0.035) |
|  | p | 0.163 | 0.646 |
|  | 95% CI | (-0.117 - 0.020) | (-0.084 - 0.052) |
| Eco impact | B | 0.025 | 0.035 |
|  | SE | (0.050) | (0.050) |
|  | p | 0.611 | 0.486 |
|  | 95% CI | (-0.073 - 0.124) | (-0.064 - 0.134) |
| Lifestyle impact | B | 0.077 | 0.023 |
|  | SE | (0.055) | (0.053) |
|  | p | 0.162 | 0.666 |
|  | 95% CI | (-0.031 - 0.186) | (-0.081 - 0.127) |
| Disrupt impact | B | 0.007 | 0.016 |
|  | SE | (0.046) | (0.045) |
|  | p | 0.869 | 0.716 |
|  | 95% CI | (-0.082 - 0.097) | (-0.072 - 0.104) |
| Citizenship effective | B | 0.520*** | 0.496*** |
|  | SE | (0.047) | (0.048) |
|  | p | 0.000 | 0.000 |
|  | 95% CI | (0.428 - 0.612) | (0.401 - 0.591) |
| Consumer effective | B | 0.154*** | 0.117*** |
|  | SE | (0.046) | (0.044) |
|  | p | 0.001 | 0.007 |
|  | 95% CI | (0.065 - 0.244) | (0.032 - 0.203) |
| Waste effective | B | -0.193*** | -0.179*** |
|  | SE | (0.053) | (0.050) |
|  | p | 0.000 | 0.000 |
|  | 95% CI | (-0.297 - -0.090) | (-0.278 - -0.081) |
| Nuclear energy use effective | B | -0.026 | 0.013 |
|  | SE | (0.026) | (0.026) |
|  | p | 0.310 | 0.621 |
|  | 95% CI | (-0.077 - 0.024) | (-0.039 - 0.065) |
| Controllable | B | 0.002 | 0.006 |
|  | SE | (0.032) | (0.030) |
|  | p | 0.962 | 0.829 |
|  | 95% CI | (-0.061 - 0.064) | (-0.052 - 0.065) |
| Perceived risk | B | 0.219*** | 0.161*** |
|  | SE | (0.040) | (0.039) |
|  | p | 0.000 | 0.000 |
|  | 95% CI | (0.139 - 0.298) | (0.085 - 0.237) |
| Trust scientists | B | 0.084*** | 0.066** |
|  | SE | (0.027) | (0.027) |
|  | p | 0.002 | 0.015 |
|  | 95% CI | (0.031 - 0.137) | (0.013 - 0.119) |
| Scientists disagree | B | 0.027 | 0.034 |
|  | SE | (0.024) | (0.025) |
|  | p | 0.263 | 0.168 |
|  | 95% CI | (-0.021 - 0.075) | (-0.014 - 0.083) |
| Past knowledge | B |  | 0.083*** |
|  | SE |  | (0.029) |
|  | p |  | 0.005 |
|  | 95% CI |  | (0.025 - 0.140) |
| Experience nature loss | B |  | 0.030 |
|  | SE |  | (0.026) |
|  | p |  | 0.249 |
|  | 95% CI |  | (-0.021 - 0.082) |
| Experience extreme events | B |  | 0.044 |
|  | SE |  | (0.028) |
|  | p |  | 0.121 |
|  | 95% CI |  | (-0.011 - 0.099) |
| Biospheric values | B |  | 0.133*** |
|  | SE |  | (0.042) |
|  | p |  | 0.002 |
|  | 95% CI |  | (0.051 - 0.216) |
| Socio-altruistic values | B |  | 0.052 |
|  | SE |  | (0.042) |
|  | p |  | 0.221 |
|  | 95% CI |  | (-0.031 - 0.134) |
| Egoistic values | B |  | -0.063** |
|  | SE |  | (0.030) |
|  | p |  | 0.039 |
|  | 95% CI |  | (-0.122 - -0.003) |
| Female+other | B |  | 0.022 |
|  | SE |  | (0.051) |
|  | p |  | 0.671 |
|  | 95% CI |  | (-0.078 - 0.121) |
| Not white | B |  | 0.073 |
|  | SE |  | (0.071) |
|  | p |  | 0.305 |
|  | 95% CI |  | (-0.067 - 0.213) |
| Age | B |  | -0.051* |
|  | SE |  | (0.027) |
|  | p |  | 0.055 |
|  | 95% CI |  | (-0.104 - 0.001) |
| Income | B |  | 0.027 |
|  | SE |  | (0.027) |
|  | p |  | 0.311 |
|  | 95% CI |  | (-0.025 - 0.080) |
| Education | B |  | 0.040 |
|  | SE |  | (0.026) |
|  | p |  | 0.123 |
|  | 95% CI |  | (-0.011 - 0.091) |
| Constant | B | 0.013 | -0.018 |
|  | SE | (0.024) | (0.036) |
|  | p | 0.585 | 0.624 |
|  | 95% CI | (-0.034 - 0.060) | (-0.089 - 0.053) |
|  |  |  |  |
| Observations |  | 602 | 602 |
| R-squared |  | 0.650 | 0.685 |
| Covariates |  | No | Yes |

Results from Ordinary Least Squares regression analyses. Robust SE (standard error) and CI (confidence interval in parentheses, B is the standardised coefficient, *** p<0.01, ** p<0.05, * p<0.1.

Table S 19: Consumer behaviours mental model:

| VARIABLES | Statistic | Model 1 | Model 1 |
| --- | --- | --- | --- |
| Internal cause | B | 0.159*** | 0.095** |
|  | SE | (0.042) | (0.045) |
|  | p | 0.000 | 0.035 |
|  | 95% CI | (0.076 - 0.243) | (0.007 - 0.183) |
| External cause | B | -0.047 | -0.025 |
|  | SE | (0.035) | (0.036) |
|  | p | 0.179 | 0.491 |
|  | 95% CI | (-0.115 - 0.021) | (-0.096 - 0.046) |
| Eco impact | B | 0.057 | 0.011 |
|  | SE | (0.053) | (0.051) |
|  | p | 0.284 | 0.826 |
|  | 95% CI | (-0.048 - 0.162) | (-0.089 - 0.112) |
| Lifestyle impact | B | -0.054 | -0.064 |
|  | SE | (0.058) | (0.057) |
|  | p | 0.349 | 0.260 |
|  | 95% CI | (-0.169 - 0.060) | (-0.175 - 0.047) |
| Disrupt impact | B | 0.133** | 0.147*** |
|  | SE | (0.052) | (0.051) |
|  | p | 0.010 | 0.004 |
|  | 95% CI | (0.031 - 0.234) | (0.047 - 0.248) |
| Citizenship effective | B | -0.021 | -0.004 |
|  | SE | (0.050) | (0.050) |
|  | p | 0.673 | 0.937 |
|  | 95% CI | (-0.120 - 0.077) | (-0.103 - 0.095) |
| Consumer effective | B | 0.719*** | 0.678*** |
|  | SE | (0.056) | (0.055) |
|  | p | 0.000 | 0.000 |
|  | 95% CI | (0.610 - 0.828) | (0.569 - 0.787) |
| Waste effective | B | -0.222*** | -0.238*** |
|  | SE | (0.064) | (0.061) |
|  | p | 0.001 | 0.000 |
|  | 95% CI | (-0.348 - -0.095) | (-0.358 - -0.119) |
| Nuclear energy use effective | B | -0.081*** | -0.029 |
|  | SE | (0.029) | (0.029) |
|  | p | 0.005 | 0.310 |
|  | 95% CI | (-0.137 - -0.024) | (-0.086 - 0.027) |
| Controllable | B | -0.019 | -0.000 |
|  | SE | (0.038) | (0.035) |
|  | p | 0.619 | 0.995 |
|  | 95% CI | (-0.093 - 0.055) | (-0.070 - 0.069) |
| Perceived risk | B | 0.140*** | 0.102*** |
|  | SE | (0.039) | (0.037) |
|  | p | 0.000 | 0.006 |
|  | 95% CI | (0.063 - 0.217) | (0.029 - 0.176) |
| Trust scientists | B | 0.030 | 0.052* |
|  | SE | (0.033) | (0.031) |
|  | p | 0.355 | 0.095 |
|  | 95% CI | (-0.034 - 0.094) | (-0.009 - 0.114) |
| Scientists disagree | B | 0.012 | 0.027 |
|  | SE | (0.030) | (0.030) |
|  | p | 0.677 | 0.356 |
|  | 95% CI | (-0.046 - 0.071) | (-0.031 - 0.085) |
| Past knowledge | B |  | 0.022 |
|  | SE |  | (0.030) |
|  | p |  | 0.457 |
|  | 95% CI |  | (-0.037 - 0.081) |
| Experience nature loss | B |  | 0.029 |
|  | SE |  | (0.029) |
|  | p |  | 0.319 |
|  | 95% CI |  | (-0.028 - 0.086) |
| Experience extreme events | B |  | 0.061** |
|  | SE |  | (0.030) |
|  | p |  | 0.040 |
|  | 95% CI |  | (0.003 - 0.119) |
| Biospheric values | B |  | 0.132*** |
|  | SE |  | (0.042) |
|  | p |  | 0.002 |
|  | 95% CI |  | (0.050 - 0.214) |
| Socio-altruistic values | B |  | 0.007 |
|  | SE |  | (0.034) |
|  | p |  | 0.842 |
|  | 95% CI |  | (-0.060 - 0.074) |
| Egoistic values | B |  | -0.128*** |
|  | SE |  | (0.033) |
|  | p |  | 0.000 |
|  | 95% CI |  | (-0.192 - -0.064) |
| Female+other | B |  | 0.089 |
|  | SE |  | (0.056) |
|  | p |  | 0.111 |
|  | 95% CI |  | (-0.021 - 0.199) |
| Not white | B |  | 0.143 |
|  | SE |  | (0.097) |
|  | p |  | 0.140 |
|  | 95% CI |  | (-0.047 - 0.333) |
| Age | B |  | 0.069** |
|  | SE |  | (0.027) |
|  | p |  | 0.012 |
|  | 95% CI |  | (0.015 - 0.122) |
| Income | B |  | -0.029 |
|  | SE |  | (0.028) |
|  | p |  | 0.302 |
|  | 95% CI |  | (-0.083 - 0.026) |
| Education | B |  | 0.064** |
|  | SE |  | (0.029) |
|  | p |  | 0.029 |
|  | 95% CI |  | (0.006 - 0.122) |
| Constant | B | 0.002 | -0.066 |
|  | SE | (0.027) | (0.040) |
|  | p | 0.927 | 0.103 |
|  | 95% CI | (-0.050 - 0.055) | (-0.145 - 0.013) |
|  |  |  |  |
| Observations |  | 602 | 602 |
| R-squared |  | 0.583 | 0.633 |
| Covariates |  | No | Yes |

Results from Ordinary Least Squares regression analyses. Robust SE (standard error) and CI (confidence interval in parentheses, B is the standardised coefficient, *** p<0.01, ** p<0.05, * p<0.1.

Table S 20: Waste behaviours mental model:

| VARIABLES | Statistic | Model 1 | Model 1 |
| --- | --- | --- | --- |
| Internal cause | B | 0.307*** | 0.255*** |
|  | SE | (0.058) | (0.058) |
|  | p | 0.000 | 0.000 |
|  | 95% CI | (0.193 - 0.420) | (0.140 - 0.370) |
| External cause | B | -0.085** | -0.056 |
|  | SE | (0.036) | (0.037) |
|  | p | 0.019 | 0.125 |
|  | 95% CI | (-0.156 - -0.014) | (-0.129 - 0.016) |
| Eco impact | B | 0.020 | 0.015 |
|  | SE | (0.056) | (0.056) |
|  | p | 0.720 | 0.789 |
|  | 95% CI | (-0.090 - 0.130) | (-0.095 - 0.125) |
| Lifestyle impact | B | 0.091 | 0.071 |
|  | SE | (0.072) | (0.070) |
|  | p | 0.202 | 0.314 |
|  | 95% CI | (-0.049 - 0.232) | (-0.067 - 0.208) |
| Disrupt impact | B | 0.016 | -0.002 |
|  | SE | (0.056) | (0.056) |
|  | p | 0.777 | 0.966 |
|  | 95% CI | (-0.094 - 0.126) | (-0.113 - 0.108) |
| Citizenship effective | B | -0.036 | -0.039 |
|  | SE | (0.050) | (0.054) |
|  | p | 0.475 | 0.474 |
|  | 95% CI | (-0.135 - 0.063) | (-0.145 - 0.068) |
| Consumer effective | B | 0.217*** | 0.197*** |
|  | SE | (0.054) | (0.055) |
|  | p | 0.000 | 0.000 |
|  | 95% CI | (0.112 - 0.322) | (0.088 - 0.306) |
| Waste effective | B | 0.220*** | 0.214*** |
|  | SE | (0.068) | (0.067) |
|  | p | 0.001 | 0.001 |
|  | 95% CI | (0.087 - 0.353) | (0.082 - 0.345) |
| Nuclear energy use effective | B | -0.043 | -0.012 |
|  | SE | (0.030) | (0.032) |
|  | p | 0.154 | 0.695 |
|  | 95% CI | (-0.101 - 0.016) | (-0.074 - 0.050) |
| Controllable | B | -0.066* | -0.063* |
|  | SE | (0.038) | (0.036) |
|  | p | 0.082 | 0.078 |
|  | 95% CI | (-0.140 - 0.008) | (-0.134 - 0.007) |
| Perceived risk | B | 0.122*** | 0.100** |
|  | SE | (0.044) | (0.045) |
|  | p | 0.005 | 0.026 |
|  | 95% CI | (0.036 - 0.207) | (0.012 - 0.187) |
| Trust scientists | B | 0.044 | 0.050 |
|  | SE | (0.039) | (0.039) |
|  | p | 0.258 | 0.206 |
|  | 95% CI | (-0.032 - 0.119) | (-0.028 - 0.127) |
| Scientists disagree | B | -0.024 | -0.015 |
|  | SE | (0.033) | (0.033) |
|  | p | 0.466 | 0.647 |
|  | 95% CI | (-0.088 - 0.040) | (-0.080 - 0.050) |
| Past knowledge | B |  | 0.053 |
|  | SE |  | (0.040) |
|  | p |  | 0.185 |
|  | 95% CI |  | (-0.026 - 0.132) |
| Experience nature loss | B |  | -0.034 |
|  | SE |  | (0.034) |
|  | p |  | 0.319 |
|  | 95% CI |  | (-0.102 - 0.033) |
| Experience extreme events | B |  | -0.004 |
|  | SE |  | (0.032) |
|  | p |  | 0.897 |
|  | 95% CI |  | (-0.066 - 0.058) |
| Biospheric values | B |  | 0.108* |
|  | SE |  | (0.055) |
|  | p |  | 0.051 |
|  | 95% CI |  | (-0.001 - 0.217) |
| Socio-altruistic values | B |  | 0.077* |
|  | SE |  | (0.044) |
|  | p |  | 0.078 |
|  | 95% CI |  | (-0.009 - 0.163) |
| Egoistic values | B |  | -0.099*** |
|  | SE |  | (0.034) |
|  | p |  | 0.004 |
|  | 95% CI |  | (-0.166 - -0.032) |
| Female+other | B |  | -0.031 |
|  | SE |  | (0.060) |
|  | p |  | 0.610 |
|  | 95% CI |  | (-0.149 - 0.087) |
| Not white | B |  | 0.197** |
|  | SE |  | (0.085) |
|  | p |  | 0.022 |
|  | 95% CI |  | (0.029 - 0.365) |
| Age | B |  | 0.062* |
|  | SE |  | (0.034) |
|  | p |  | 0.072 |
|  | 95% CI |  | (-0.006 - 0.129) |
| Income | B |  | 0.008 |
|  | SE |  | (0.031) |
|  | p |  | 0.790 |
|  | 95% CI |  | (-0.052 - 0.068) |
| Education | B |  | 0.047* |
|  | SE |  | (0.027) |
|  | p |  | 0.077 |
|  | 95% CI |  | (-0.005 - 0.099) |
| Constant | B | -0.001 | -0.012 |
|  | SE | (0.030) | (0.043) |
|  | p | 0.987 | 0.790 |
|  | 95% CI | (-0.059 - 0.058) | (-0.097 - 0.074) |
|  |  |  |  |
| Observations |  | 602 | 602 |
| R-squared |  | 0.493 | 0.531 |
| Covariates |  | No | Yes |

Results from Ordinary Least Squares regression analyses. Robust SE (standard error) and CI (confidence interval in parentheses, B is the standardised coefficient, *** p<0.01, ** p<0.05, * p<0.1.

Table S 21: Nuclear energy use mental model:

| VARIABLES | Statistic | Model 1 | Model 1 |
| --- | --- | --- | --- |
| Internal cause | B | 0.070 | 0.045 |
|  | SE | (0.058) | (0.056) |
|  | p | 0.227 | 0.425 |
|  | 95% CI | (-0.043 - 0.183) | (-0.066 - 0.156) |
| External cause | B | -0.040 | -0.009 |
|  | SE | (0.040) | (0.039) |
|  | p | 0.317 | 0.812 |
|  | 95% CI | (-0.118 - 0.038) | (-0.086 - 0.067) |
| Eco impact | B | -0.085* | -0.066 |
|  | SE | (0.047) | (0.051) |
|  | p | 0.073 | 0.194 |
|  | 95% CI | (-0.177 - 0.008) | (-0.167 - 0.034) |
| Lifestyle impact | B | 0.027 | 0.026 |
|  | SE | (0.060) | (0.060) |
|  | p | 0.656 | 0.667 |
|  | 95% CI | (-0.090 - 0.144) | (-0.093 - 0.145) |
| Disrupt impact | B | 0.052 | 0.013 |
|  | SE | (0.053) | (0.056) |
|  | p | 0.330 | 0.811 |
|  | 95% CI | (-0.052 - 0.156) | (-0.097 - 0.123) |
| Citizenship effective | B | -0.103** | -0.110** |
|  | SE | (0.049) | (0.053) |
|  | p | 0.036 | 0.039 |
|  | 95% CI | (-0.199 - -0.007) | (-0.214 - -0.005) |
| Consumer effective | B | 0.067 | 0.075 |
|  | SE | (0.051) | (0.052) |
|  | p | 0.188 | 0.152 |
|  | 95% CI | (-0.033 - 0.166) | (-0.028 - 0.178) |
| Waste effective | B | -0.137*** | -0.127** |
|  | SE | (0.051) | (0.054) |
|  | p | 0.008 | 0.018 |
|  | 95% CI | (-0.238 - -0.036) | (-0.233 - -0.022) |
| Nuclear energy use effective | B | 0.736*** | 0.735*** |
|  | SE | (0.026) | (0.028) |
|  | p | 0.000 | 0.000 |
|  | 95% CI | (0.685 - 0.787) | (0.679 - 0.791) |
| Controllable | B | 0.003 | 0.004 |
|  | SE | (0.036) | (0.035) |
|  | p | 0.932 | 0.901 |
|  | 95% CI | (-0.067 - 0.074) | (-0.064 - 0.072) |
| Perceived risk | B | 0.030 | 0.030 |
|  | SE | (0.042) | (0.041) |
|  | p | 0.471 | 0.473 |
|  | 95% CI | (-0.052 - 0.111) | (-0.052 - 0.111) |
| Trust scientists | B | 0.037 | 0.037 |
|  | SE | (0.033) | (0.034) |
|  | p | 0.266 | 0.272 |
|  | 95% CI | (-0.028 - 0.102) | (-0.029 - 0.103) |
| Scientists disagree | B | 0.044 | 0.058* |
|  | SE | (0.029) | (0.029) |
|  | p | 0.134 | 0.051 |
|  | 95% CI | (-0.014 - 0.101) | (-0.000 - 0.115) |
| Past knowledge | B |  | 0.092*** |
|  | SE |  | (0.035) |
|  | p |  | 0.008 |
|  | 95% CI |  | (0.024 - 0.160) |
| Experience nature loss | B |  | 0.003 |
|  | SE |  | (0.034) |
|  | p |  | 0.929 |
|  | 95% CI |  | (-0.064 - 0.071) |
| Experience extreme events | B |  | 0.040 |
|  | SE |  | (0.033) |
|  | p |  | 0.218 |
|  | 95% CI |  | (-0.024 - 0.105) |
| Biospheric values | B |  | -0.010 |
|  | SE |  | (0.042) |
|  | p |  | 0.811 |
|  | 95% CI |  | (-0.093 - 0.073) |
| Socio-altruistic values | B |  | -0.013 |
|  | SE |  | (0.038) |
|  | p |  | 0.731 |
|  | 95% CI |  | (-0.088 - 0.062) |
| Egoistic values | B |  | -0.011 |
|  | SE |  | (0.032) |
|  | p |  | 0.722 |
|  | 95% CI |  | (-0.074 - 0.052) |
| Female+other | B |  | -0.146** |
|  | SE |  | (0.063) |
|  | p |  | 0.022 |
|  | 95% CI |  | (-0.270 - -0.021) |
| Not white | B |  | 0.058 |
|  | SE |  | (0.084) |
|  | p |  | 0.495 |
|  | 95% CI |  | (-0.108 - 0.223) |
| Age | B |  | 0.048 |
|  | SE |  | (0.031) |
|  | p |  | 0.120 |
|  | 95% CI |  | (-0.012 - 0.108) |
| Income | B |  | -0.031 |
|  | SE |  | (0.032) |
|  | p |  | 0.327 |
|  | 95% CI |  | (-0.094 - 0.031) |
| Education | B |  | 0.011 |
|  | SE |  | (0.029) |
|  | p |  | 0.695 |
|  | 95% CI |  | (-0.045 - 0.068) |
| Constant | B | 0.009 | 0.067 |
|  | SE | (0.028) | (0.045) |
|  | p | 0.761 | 0.141 |
|  | 95% CI | (-0.046 - 0.063) | (-0.022 - 0.156) |
|  |  |  |  |
| Observations |  | 602 | 602 |
| R-squared |  | 0.535 | 0.554 |
| Covariates |  | No | Yes |

Results from Ordinary Least Squares regression analyses. Robust SE (standard error) and CI (confidence interval in parentheses, B is the standardised coefficient, *** p<0.01, ** p<0.05, * p<0.1.
